# miR-193b and miR-30c-1^*^ inhibit, whereas miR-576-5p enhances melanoma cell invasion *in vitro*

**DOI:** 10.18632/oncotarget.25986

**Published:** 2018-08-21

**Authors:** Theresa Kordaß, Claudia E.M. Weber, David Eisel, Antonino A. Pane, Wolfram Osen, Stefan B. Eichmüller

**Affiliations:** ^1^ GMP and T Cell Therapy Unit, German Cancer Research Center (DKFZ), Heidelberg, Germany; ^2^ Faculty of Biosciences, University Heidelberg, Heidelberg, Germany

**Keywords:** melanoma, miRNA, invasion, BCL9, MCL1

## Abstract

In cancer cells, microRNAs (miRNAs) are often aberrantly expressed resulting in impaired mRNA translation. In this study we show that miR-193b and miR-30c-1^*^ inhibit, whereas miR-576-5p accelerates invasion of various human melanoma cell lines. Using Boyden chamber invasion assays the effect of selected miRNAs on the invasive capacity of various human melanoma cell lines was analyzed. Upon gene expression profiling performed on transfected A375 cells, CTGF, THBS1, STMN1, BCL9, RAC1 and MCL1 were identified as potential targets. For target validation, qPCR, Western blot analyses or luciferase reporter assays were applied. This study reveals opposed effects of miR-193b / miR-30c-1^*^ and miR-576-5p, respectively, on melanoma cell invasion and on expression of BCL9 and MCL1, possibly accounting for the contrasting invasive phenotypes observed in A375 cells transfected with these miRNAs. The miRNAs studied and their targets identified fit well into a model proposed by us explaining the regulation of invasion associated genes and the observed opposed phenotypes as a result of networked direct and indirect miRNA / target interactions. The results of this study suggest miR-193b and miR-30c-1^*^ as tumor-suppressive miRNAs, whereas miR-576-5p appears as potential tumor-promoting oncomiR. Thus, miR-193b and miR-30c-1^*^ mimics as well as antagomiRs directed against miR-576-5p might become useful tools in future therapy approaches against advanced melanoma.

## INTRODUCTION

Among all skin cancer types, melanoma has the highest mortality rate showing rising incidence [1]. Indeed, effective therapy is currently only available for early stage melanoma. In the case of metastatic melanoma, however, standard therapies like chemo- and radiotherapy have yielded only moderate success, so far. Thus, novel therapeutic strategies improving the clinical outcome of patients suffering from advanced melanoma are desperately needed. A promising starting point might be to modulate expression of cellular targets linked to regulation of melanoma progression and tumor cell invasion. In this respect, miRNAs, representing a group of small noncoding RNA molecules regulating protein expression at post-transcriptional level [2] by targeting specific mRNA molecules, appear of particular interest [3]. Of note, miRNAs can act as switches in differentiation processes [4]. Thus, expression levels of miR-302a-5p which selectively occur in pluripotent stem cells were found to decline during stem cell differentiation [5]. Moreover, miRNAs have been shown to inhibit tumor cell migration and invasion as described for miR-211 in hepatocellular carcinoma [6] and for miR-339-3p in melanoma [7], respectively. Similarly, miR-101 was found to decrease invasion in various cancer types such as glioblastoma [8], bladder cancer [9], hepatocellular carcinoma [10] and melanoma [11]. An example for an invasion-promoting miRNA is miR-222, shown to increase migration and invasion in colorectal cancer [12]. Also miR-10b was shown to increase migration and invasion of hepatocellular carcinoma cells by directly targeting tumor suppressor cell adhesion molecule 2 (CADM2) [13]. miRNA expression profiles are often altered in cancer cells [14], hence miRNA profiling has been used for tumor classification and as prognostic tool to predict patients’ survival or clinical response to administered drugs. Thus, high expression levels of miR-155 and low let-7a-2 levels were found to be associated with poor survival of lung cancer patients [15]. Moreover, determination of specific miRNA signatures can be used to discriminate tumor entities and to distinguish tumor from healthy tissue [4, 16] as reported, for example, in a study showing that a signature of 30 miRNAs allowed discrimination between normal intrahepatic bile ducts (NIBD) and intrahepatic cholangiocarcinoma (ICC) with 100% accuracy [17]. Furthermore, aberrant miRNA profiles present in blood can be used as biomarkers, enabling diagnosis of cancer at early, pre-symptomatic stages, as shown for miR-16, whose reduced plasma levels were found to be associated with hepatocellular carcinoma (HCC) [17].

Interestingly, certain miRNAs promoting tumor progression called oncomiRs were found overexpressed in tumors and moreover, a huge array of miRNAs has been established that can affect the vulnerability of tumor cells to immune surveillance [18]. In analogy to approaches targeting oncogenes encoding growth factors such as human epidermal growth factor receptor 2 (HER2) in breast cancer or protein kinases, e.g. BRAF in melanoma, oncogenic miRNAs as well might serve as therapeutic targets that could be silenced through application of antisense oligomers, called antagomiRs [19, 20]. However, intracellular delivery and specific mRNA targeting as well as prevention of side-effects still constitute a major challenge. Thus until today, only three miRNA inhibitors have entered phase I or phase II clinical trials, respectively [21]. One of these antagomiRs designed to inhibit liver specific miR-122 involved in infection of Hepatitis C virus (HCV) resulted in reduced virus load [22, 23]. Besides the use of antagomiRs to inhibit oncogenic miRNAs, application of miRNA mimics of tumor suppressive miRNAs has also been considered as a therapeutic option. Three miRNA mimics are currently tested in clinical phase I trials [21]. One of these, mimic miR-34, is applied to treat metastatic liver cancer [21]. As a tumor suppressor, this miRNA targets various oncogenic pathways thereby inhibiting cancer cell growth and proliferation [22, 24].

In our study presented here, we investigate in detail the functional impact of three miRNAs (miR-30c-1^*^, miR-193b and miR-576-5p) on melanoma cell invasion and reveal the underlying modes of action.

## RESULTS

### miR-576-5p enhances, whereas miR-193b and miR-30c-1^*^ inhibit invasion of various melanoma cell lines

**Figure 1:**
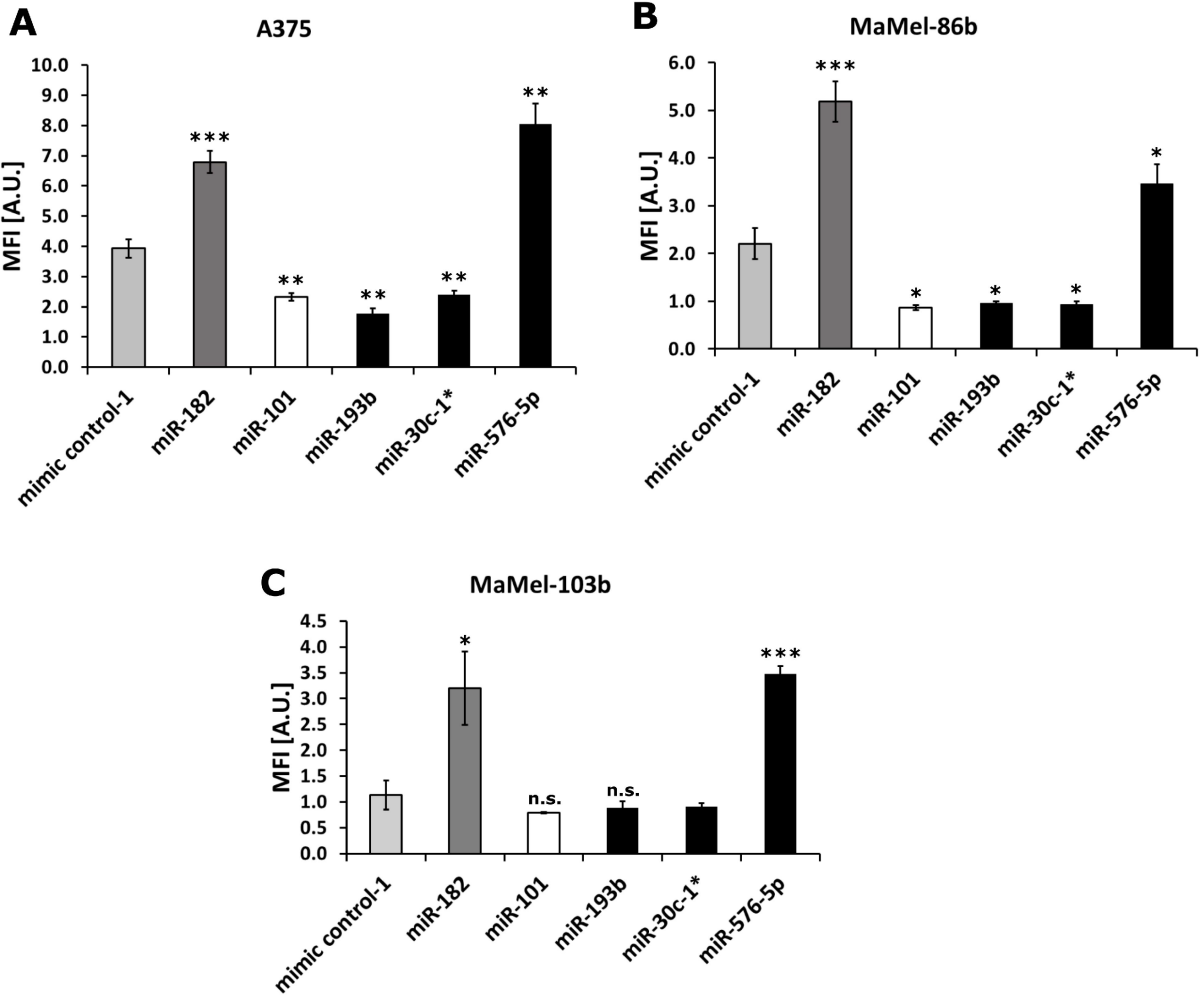
Melanoma cell invasion affected by miR-576-5p, miR-193b and miR-30c-1^*^ For three melanoma cell lines A375 (**A**), MaMel-86b (**B**) and MaMel-103b (**C**) Matrigel-based Boyden chamber invasion assays were performed after transfection with 50 nM miRNA. Two days post transfection cells were seeded in inner wells of Boyden chamber plate and number of invaded cells was measured 24 h later. Fluorescence intensity reflects the number of invaded cells. Three technical replicates were performed per condition and mean fluorescence intensity (MFI) ± SD are displayed. ^*^p < 0.05, ^**^p < 0.01 ^***^p < 0.001.

First, the effect of miRNAs miR-30c-1^*^, miR-193b and miR-576-5p shown to affect the invasive capacity of A375 melanoma cells [7] was confirmed on additional melanoma cell lines including A375 as reference. Therefore, MaMel-86b, MaMel-103b, and A375 cells were transfected with 50 nM miRNA and subsequently analyzed in Matrigel-based invasion assays. miR-182 [17, 29] and miR-101 [10, 30] served as positive and negative controls, respectively. As shown in Figure 1A, miR-576-5p (fold change FC = 2.1) enhanced invasion of A375 cells to similar extent as miR-182 (FC = 1.7). Conversely, miR-30c-1^*^ (FC = 0.61) and miR-193b (FC = 0.45) diminished the invasive capacity comparable to miR-101 (FC = 0.59). Similar effects were observed in transfected MaMel-86b cells with miR-576-5p leading to enhanced invasion (FC = 1.6), whereas miR-30c-1^*^ (FC = 0.4) and miR-193b (FC = 0.4) induced the opposite effect (Figure 1B). MaMel-103b cells transfected with miR-576-5p showed invasive capacity that was enhanced (FC = 3.1) to an extent comparable to that caused by the positive control miR-182 (FC = 2.8), whereas no reduction in invasiveness was observed with miR-193b and miR-30c-1^*^ (Figure 1C). However, as the negative control miR-101 failed to significantly reduce invasion of MaMel-103b cells as well, we suggest that this cell line’s invasive capacity is too low *per se* to be reduced even further.

### miR-576-5p and miR-30c-1^*^ / miR-193b show opposed effects on melanoma cell proliferation

**Figure 2:**
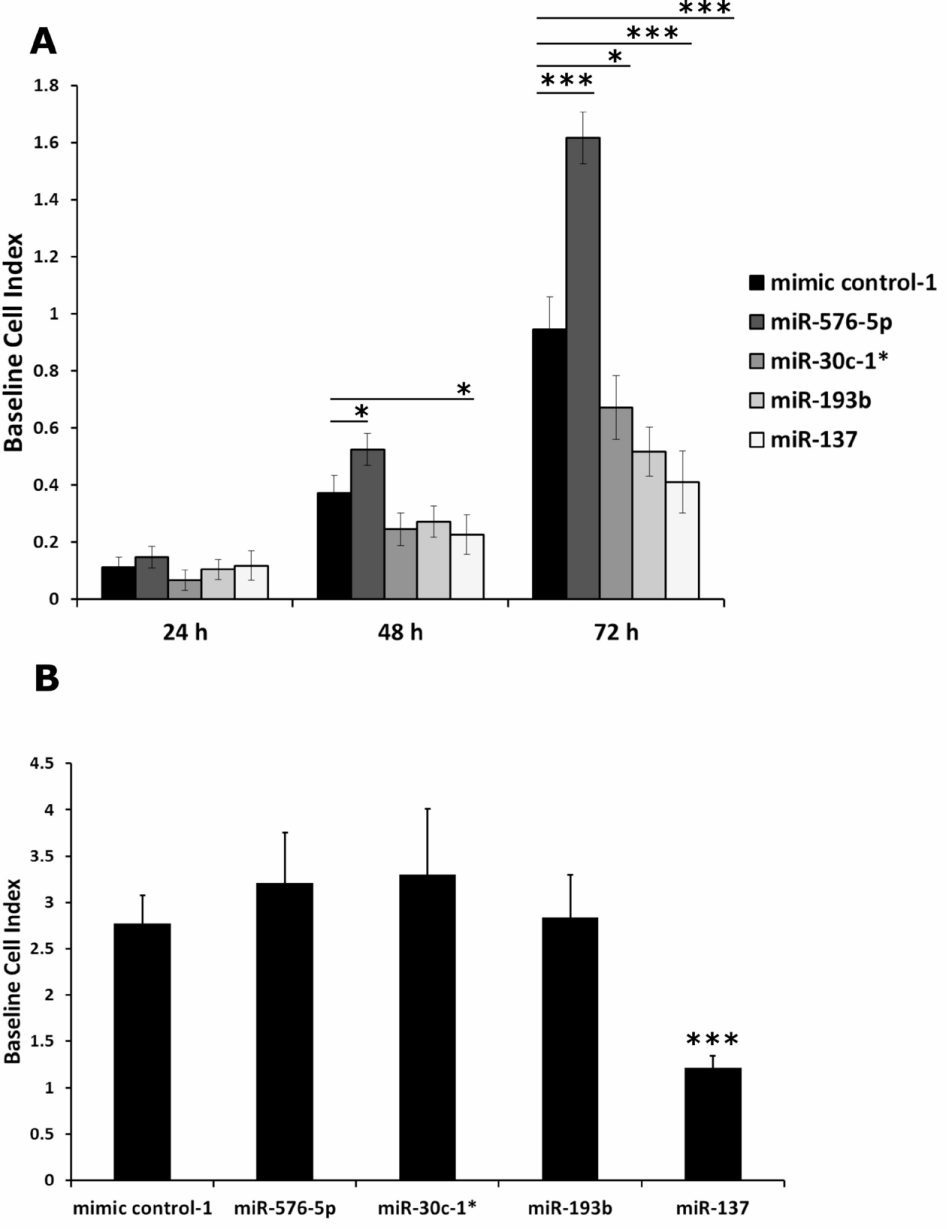
Impedance based proliferation assay performed with miRNA transfected A375 cells Proliferation of A375 cells transfected with 50 nM miRNA was monitored by measuring impedance which is proportional to the number of adherent cells, expressed as Baseline Cell Index. Samples transfected with miR-137 severed as a control for reduced proliferation. (**A**) Impact on proliferation was most pronounced after 72 h: miR-576-5p promotes, whereas miR-137, miR-30c-1^*^ and miR-193b reduced the proliferative capacity of A375 cells. (**B**) Separate proliferation assay to determine possible impact on invasion assay. A375 cells (5·10^4^ per well) were seeded 48 h after transfection and measurement was performed for 24 h. Only A375 cells transfected with miR-137 showed significantly altered proliferation. Three biological replicates were performed per condition and mean values ± SD are displayed. ^*^p < 0.05, ^***^p < 0.001.

The invasive activity of the transfected melanoma cell lines determined by Matrigel assays above might have been caused at least in part by differences in viability and or proliferative capacity, rather than by induction of invasive functions. Thus, viability tests and proliferation assays were performed to clarify this issue. As shown in Supplementary Figure 2, even 72 hours after transfection none of the miRNAs showed a significant effect on cell viability on any of the cell lines tested. Further, monitoring the proliferation of miRNA transfected A375 cells revealed that none of the miRNAs affected cell proliferation within 24 h after transfection (Figure 2A; Supplementary Figure 3). This was different when miRNA mediated effects on proliferation were analyzed at later time points. Hence 48 h after transfection, proliferation of cells transfected with miR-30c-1^*^ or miR-193b was reduced to a similar level as caused by transfection of the negative control miR-137 [31]. In contrast, miR-576-5p transfected A375 cells showed increased proliferation compared to cells transfected with mimic control-1. This opposed effect was even more pronounced in A375 cells analyzed 72 h post transfection. Again, transfection of miR-576-5p strongly enhanced proliferation of A375 cells, whereas miR-30c-1^*^ and miR-193b lead to a significant reduction of proliferation.

In order to assess whether an altered proliferation rate might impact the outcome of our invasion assays, we measured proliferation in a separate experiment using the same parameters as applied in the invasion assay. Briefly, 48 h after transfection a defined number (i.e. 5·10^4^) of miRNA transfected A375 cells was seeded into a 96 well E-plate and proliferation was measured using an impedance based read out system. None of the miRNAs showed a significant impact on proliferation of transfected A375 cells during 24 h of cultivation, except for miR-137 which served as control with known inhibitory effect on proliferation (Figure 2B). In addition, cell lines MaMel-103b and MaMel-86b were tested under the same conditions using the XTT proliferation assay and no effect by either of the three miRNAs was found (not shown).

### Expression of miR-576-5p correlates with the invasive potential of various human melanoma cell lines

**Figure 3:**
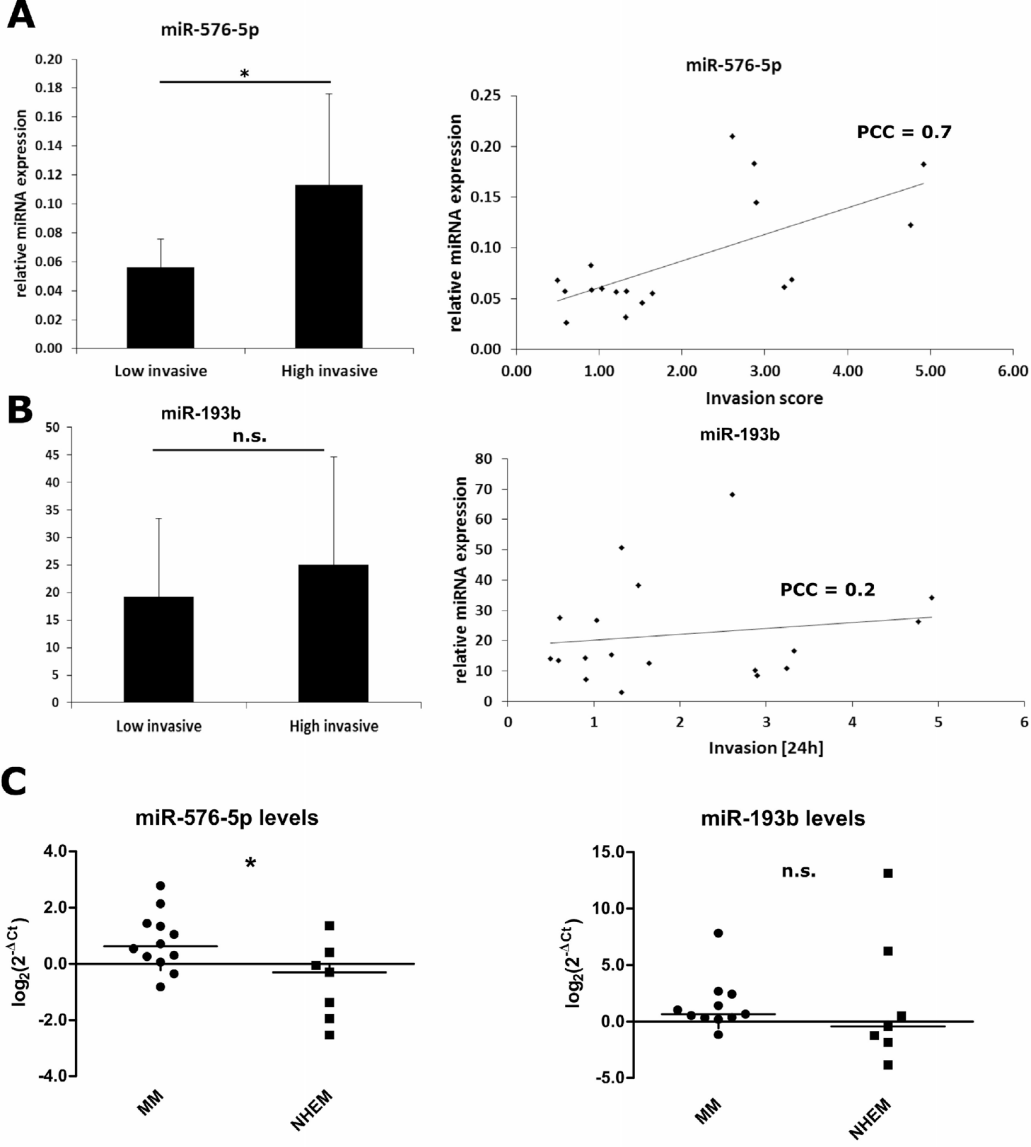
Expression of miR-576-5p, but not miR-193b correlates positively with the invasive potential of melanoma cell lines Expression of miR-576-5p (**A**) and miR-193b (**B**) was quantified in 18 human melanoma cell lines by qPCR. Endogenous expression of miR-30c-1^*^ was below detection limit. For all cell lines basal invasion of untreated cells was measured and correlated with miR-576-5p or miR-193b expression, respectively. Based on the median invasion score, cell lines were categorized as weakly invasive (*n* = 9) and highly invasive (*n* = 9), respectively. The expression level of miR-576-5p was significantly enhanced (*p* = 0.018, FC = 2) in the highly invasive subgroup. Furthermore, miR-576-5p expression showed a positive correlation with the invasion score (PCC = 0.7, *p* = 0.003). No significant correlation between miR-193b expression level and invasion could be observed. (**C**) miRNA expression levels of miR-576-5p and miR-193b were determined for metastatic melanoma samples (MM) and normal human melanocytes (NHEM) with qPCR. For miR-576-5p a significant higher expression (*p* = 0.02) in MM could be observed compared to NHEM. For miR-193b levels there was no significant difference between MM and NHEM.

Having shown the modulating effects of miR-576-5p, miR-193b and miR-30c-1^*^ on melanoma cell invasion, we screened a panel of 18 human melanoma cell lines for expression of these mRNAs and correlated the expression levels to the invasive capacity of the respective cell lines. The invasion scores are compiled in Supplementary Table 3. Based on the median invasion score of all cell lines tested (Median Invasion score = 1.42), subgroups with high vs low invasive capacity were defined. As depicted in Figure 3A, the expression level of miR-576-5p showed a positive correlation with invasive capacity (PCC = 0.7, *p* = 0.003) and was enhanced twofold in the highly invasive group (Figure 3A left) (*p* = 0.018). On the other hand, no correlation between miR-193b expression levels and invasiveness of human melanoma cell lines (Figure 3B) was observed. Expression levels miR-30c-1^*^ were below detection limit and could not be analyzed (not shown). Notably, expression levels of miR-576-5p appeared significantly enhanced in melanoma cell lines too when compared to healthy melanocytes, whereas expression levels of miR-193b did not show significant differences relative to normal melanocytes (Figure 3C). Together these data demonstrate a positive correlation between miR-576-5p expression levels and invasive capacity of melanoma cell lines *in vitro*.

### Gene expression profiles can explain different invasive phenotypes

**Figure 4:**
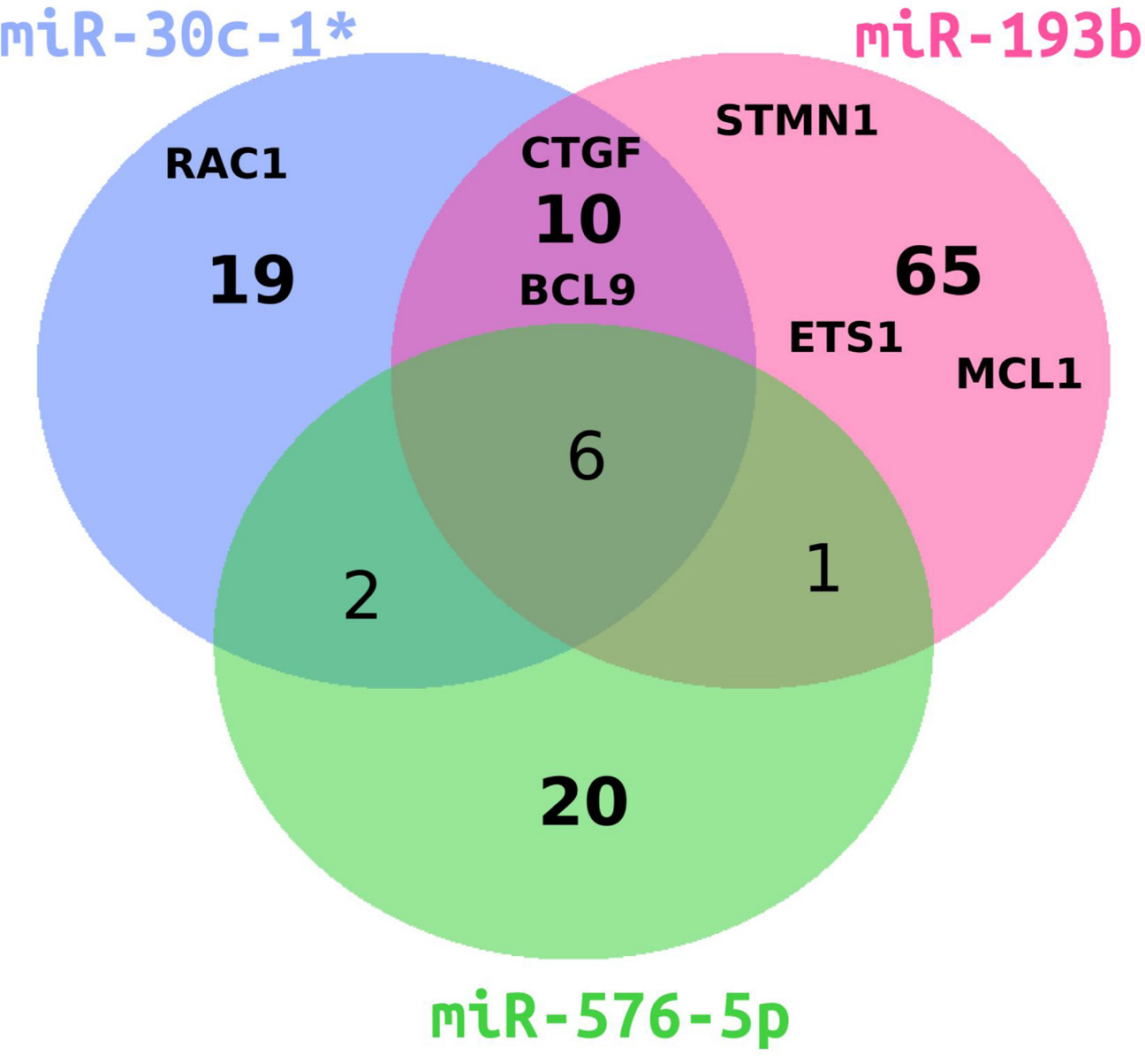
Venn diagram of differentially downregulated genes in miRNA-transfected A375 cells Overlap of significantly down-regulated genes with a fold change smaller than 0.7 for miR-193b (magenta), miR-30c-1^*^ (blue) or miR-576-5p (green) is shown. Genes further analyzed in this study are indicated by abbreviations.

To understand which changes in gene expression mediated by the miRNAs of investigation would drive the invasiveness of melanoma cell lines, we performed gene expression profiling of A375 cells individually transfected with miR-30c-1^*^, miR-193b and miR-576-5p following the workflow shown in Supplementary Figure 1. Transfection with miR-193b resulted in 3,341 differentially expressed genes (Supplementary Spread Sheet “Differential_Genes.xlsx”), which is the highest number of affected genes among the three miRNAs tested. Transfection with miR-30c-1^*^ resulted in 1,789 differentially expressed genes and transfection with miR-576-5p induced the lowest change of the transcriptome with 1,479 differentially expressed genes. The overlap of genes affected by all three miRNAs consisted of 365 genes. Each miRNA yielded at least 43% uniquely affected genes. In Figure 4, genes significantly down-regulated with a fold-change (FC) below 0.7 are depicted. The majority (51–79%) of down-regulated genes was unique for the respective miRNA. In the following, genes with a FC < 0.7 are considered as down-regulated, whereas genes with a FC > 1.3 are judged as up-regulated. Differentially expressed genes maximally down- or up-regulated are listed in Tables 1 and 2, respectively.

**Table 1:** Genes showing reduced differential expression in A375 cells after transfection with miR-193b, miR30c-1^*^ or miR-576-5p

miR-193b		miR-30c-1^*^		miR-576-5p	
Gene	FC	Gene	FC	Gene	FC
ARHGDIB	0.50	HIST1H2BK	0.50	HIST1H2BK	0.55
WSB2	0.53	THBS1	0.57	TGFB1	0.56
STMN1	0.56	BCL9	0.59	SCG5	0.58
C9orf30	0.56	SCG5	0.60	ADAM19	0.62
TPM1	0.57	LOC652595	0.61	NRP1	0.63
THBS1	0.58	APP	0.62	LOC728069	0.63
STARD7	0.58	RAC1	0.63	CDKN1C	0.64
EFEMP1	0.59	NTM	0.63	NTM	0.65
DCAF7	0.60	CPA4	0.63	ITGA5	0.65
PTPRF	0.60	CYR61	0.63	TMEM45A	0.65
SLC35D2	0.60	LOC392437	0.63	LOC652595	0.65
CTGF	0.60	IL7R	0.64	LOC148430	0.65
ETS1	0.60	CTGF	0.65	LOC653888	0.66

FC: Fold changes relative to mimic control-1 transfected samples.

**Table 2:** Genes showing enhanced differential expression in A375 cells after transfection with miR-193b, miR30c-1^*^ or miR-576-5p

miR-193b		miR-30c-1^*^		miR-576-5p	
Gene	FC	Gene	FC	Gene	FC
TCN1	6.07	AMMECR1	1.84	LOC647315	1.79
IGFBP3	2.86	NDUFB5	1.73	MGC24103	1.76
RDH5	2.81	EWSR1	1.73	STMN3	1.76
IGFBP3	2.76	RPS23	1.65	DKK1	1.76
KRT6B	2.39	MRPS35	1.65	NDUFB5	1.75
STC1	2.27	N-PAC	1.62	DPYSL3	1.66
TAGLN3	2.02	ARNT2	1.58	EWSR1	1.65
SLC22A4	2.02	ATP9A	1.56	LOC729009	1.63
VGF	1.98	TPD52L2	1.56	MIR1978	1.58
IL1A	1.97	CANX	1.55	LOC728499	1.57
CD55	1.95	UHMK1	1.54	CBX2	1.55
SPP1	1.94	LOC100129098	1.54	ARL5A	1.55
CTXN1	1.93	LOC100133005	1.52	FST	1.55

FC: Fold changes relative to mimic control-1 transfected samples.

Next, we performed pathway analyses of differentially expressed genes to determine possible alterations in given gene expression networks. For this purpose two methods were applied. First, Gene Set Enrichment Analysis (GSEA) was performed using the R piano package [32]. For this method, all genes are included and ranked according to their *p*-values. The algorithm then determines enriched gene sets taking up-regulated as well as down-regulated genes into account. The GSEA analysis revealed that metabolic pathways were enriched for all three miRNAs analyzed (Supplementary Table 4). Notably, the focal adhesion pathway [33] was enriched for all three miRNAs as well, which is of particular interest with regard to invasion. Also, the pathways of adherent junctions and extracellular matrix (ECM)-receptor interaction which are enriched for miR-576-5p transfected A375 samples are known to be involved in invasion [34, 35]. Importantly, the TGF-*β* signaling pathway was enriched for all three miRNAs as well, and in cells transfected with miR-30c-1^*^ we found the p53 signaling pathway to be enriched.

As a second approach, genes significantly down- (FC < 0.7) or up-regulated (FC > 1.3) were entered into the online platform DAVID 6.7 [36]. The enriched pathways revealed are presented in Supplementary Table 5. Considering down-regulated genes, the cell adhesion pathway was enriched for all three miRNAs. This result is in accordance with the outcome of the GSEA analysis, confirming that focal adhesion was enriched for all three miRNAs. More precisely, for miR-193b and miR-30c-1^*^ transfected samples, positive regulation of cell adhesion was diminished, possibly reflecting the reduced invasive phenotype observed for these two miRNAs (Figure 1).

### Selection of potential targets

From the list of differentially downregulated genes (Table 1), six candidates were selected for further analysis. These included Connective Tissue Growth Factor (CTGF), because of its selective downregulation in miR-30c-1^*^ and miR-193b transfected samples, potentially explaining the reduced invasiveness upon transfection of miR-30c-1^*^ and miR-193b, as well as the enhanced invasion observed with miR-576-5p transfected cells. Thrombospondin 1 (THBS1) was selected, since it was significantly downregulated by all three miRNAs, similar to Ras-related C3 botulinum toxin substrate 1 (RAC1), the latter representing a potential target for cancer therapy [37], as activating RAC1 mutations can drive tumor progression [38]. Furthermore, Stathmin 1 (STMN1) was selected as it was found uniquely down-regulated in miR-193b transfected cells, and due to the fact that STMN1 represents a potential target for melanoma therapy [39]. Of note, Li *et al.* recently showed that miR-193b directly targets STMN1, thus confirming our result [40]. A gene down-regulated in miR-30c-1^*^ (FC = 0.59) and in miR-193b (FC = 0.74) transfected cells was B-cell CLL/lymphoma 9 protein (BCL9), known to enhance invasion and to promote tumor progression [41]. Downregulation of BCL9 could explain the observed reduced invasiveness of A375 cells after transfection with miR-30c-1^*^ or miR-193b. Finally, we included induced myeloid leukemia cell differentiation protein (MCL1) in our following analyses, since it was recently identified as a direct target of miR-339-3p [7].

**Figure 5:**
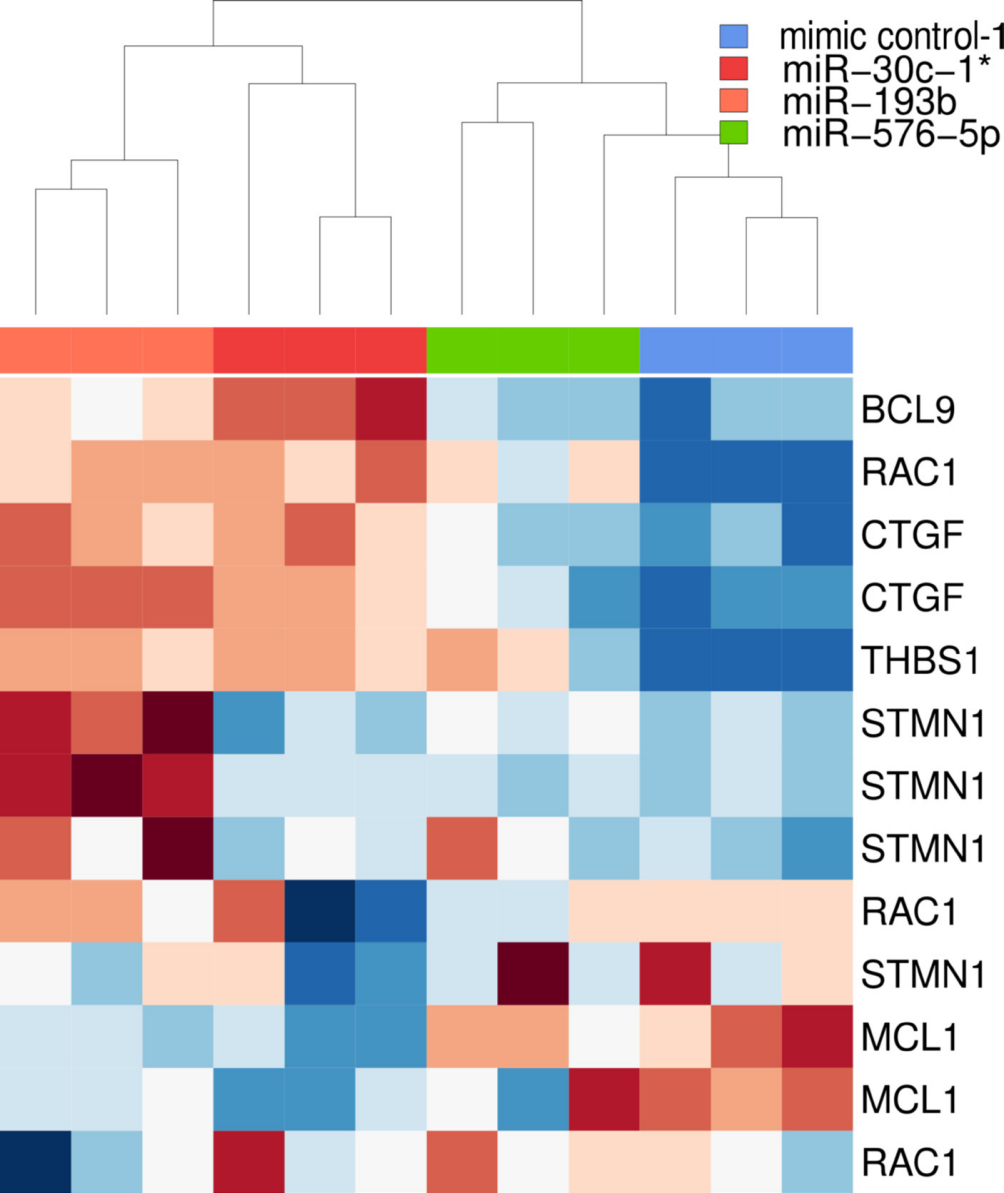
Heatmap based on downregulated genes selected for further analysis The unsupervised clustering was based on average-linkage and euclidean distance. Gene expression data were log_2_ transformed and z-normalized gene-wise. Red represents low expression, white average expression and blue high expression. Genes used for clustering are listed at the right side. A given gene can occur multiple times due to application of different probes specific for the same gene.

The selected genes were then subjected to unsupervised hierarchical cluster analysis based on their expression level to examine whether this panel of genes was sufficient to group the three biological replicates of each condition (mimic control-1, miR-30c-1^*^, miR-193b and miR-576-5p), and whether samples sharing a similar phenotype (miR-30c-1^*^ & miR-193b vs. mimic control-1 & miR-576-5p) could thereby be discriminated (Figure 5). The clustering based on average-linkage and euclidean distance allowed clear discrimination of the two groups. Interestingly, this categorization did not apply when considering the entire set of differentially expressed genes (see Supplementary Figure 4).

### Verification of novel miRNA targets on mRNA and protein level

**Figure 6:**
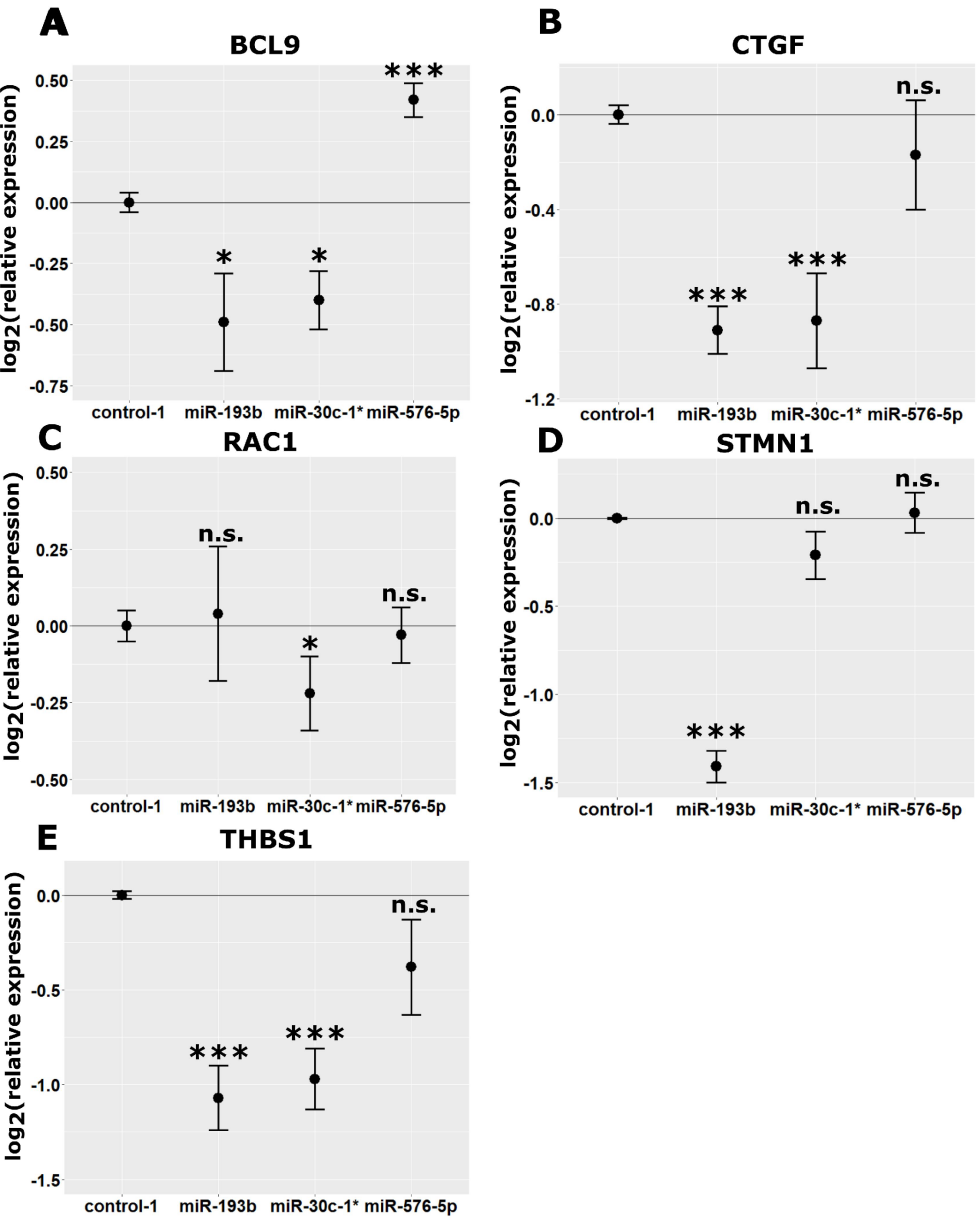
Modulation of target mRNA expression in miRNA transfected A375 cells A375 cells were transfected with 50 nM mimic control-1, miR-30c-1^*^, miR-193b or miR-576-5p. After RNA isolation, cDNA was generated and qPCR was performed. The same samples were used for microarray analysis. Expression levels of BCL9 (**A**), CTGF (**B**), RAC1 (**C**), STMN1 (**D**) and THBS1 (**E**) were normalized to GAPDH. Mean values of three biological replicates are shown ± SD. Conditions were compared to mimic control-1 and significance was assessed with two-sample Student’s *T*-tests. ^*^p < 0.05, ^**^p < 0.01, ^***^p < 0.005.

The effect of miR-30c-1^*^, miR-193b and miR-576-5p on putative target genes was verified on posttranscriptional level by qPCR, described as superior method to quantify changes in gene expression levels [42]. The analyses were performed on the same samples used for gene expression profiling. Analysis of the microarray data revealed significantly down-regulated BCL9 levels in miR-30c-1^*^ and miR-193b transfected A375 cells (Supplementary Spread Sheet “Differential_Genes.xlsx”), which was in line with qPCR results shown in Figure 6. Cells transfected with miR-193b exhibited the strongest downregulation of BCL9 expression (log_2_(FC) = –0.49; Figure 6A). Similar downregulation of BCL expression was observed in samples from miR-30c-1^*^ transfected cells (log_2_(FC) = –0.40; Figure 6A). Of note, no significant effect of miR-576-5p on BCL9 expression could be observed in microarray analyses, which is in contrast to the qPCR results that showed a significant increase in BCL9 expression levels (log_2_(FC) = 0.42, Figure 6A). Regarding CTGF expression, our qPCR data confirmed the microarray results showing that CTGF levels were selectively downregulated by miR-193b (log_2_(FC) = –0.91, Figure 6B) and miR-30c-1^*^ (log_2_(FC) = –0.87, Figure 6B). With respect to RAC1 expression, the microarray data obtained could not entirely be confirmed by qPCR, since only miR-30c-1^*^ transfected cells showed a significant reduction in RAC1 expression levels (log_2_(FC) = −0.22, Figure 6C).

In accordance with the microarray data, STMN1 expression was found down-regulated selectively in miR-193b transfected cells (log_2_(FC) = –1.41, Figure 6D). For miR-30c-1^*^, only a tendency towards reduced STMN1 expression was observed (log_2_(FC) = –0.21, Figure 6D). No effect on STMN1 expression was detected upon transfection with miR-576-5p (log_2_(FC) = 0.03, Figure 6D). According to the microarray data, THBS1 expression was significantly downregulated by all three miRNAs, however when used for qPCR, the same samples showed significant reduction in expression of this gene only upon transfection with miR-193b (log_2_(FC) = –0.97, Figure 6E) or miR-30c-1^*^ (log_2_(FC) = –1.07, Figure 6E).

In order to test, whether the observed effects of the investigated miRNAs on the selected targets are not cell line specific, we repeated the qPCR measurements in MaMel-103b and MaMel-86b after miRNA mimic transfection (see Supplementary Figures 5 and 6, respectively). The effects of all three miRNAs on CTGF and STMN1 expression could be fully confirmed in MaMel-103b and MaMel-86b. Downregulation of BCL9 by miR-30c-1^*^ and upregulation by miR-576-5p could be also confirmed, whereas regulation of RAC1 and THBS1 was ambiguous.

**Figure 7:**
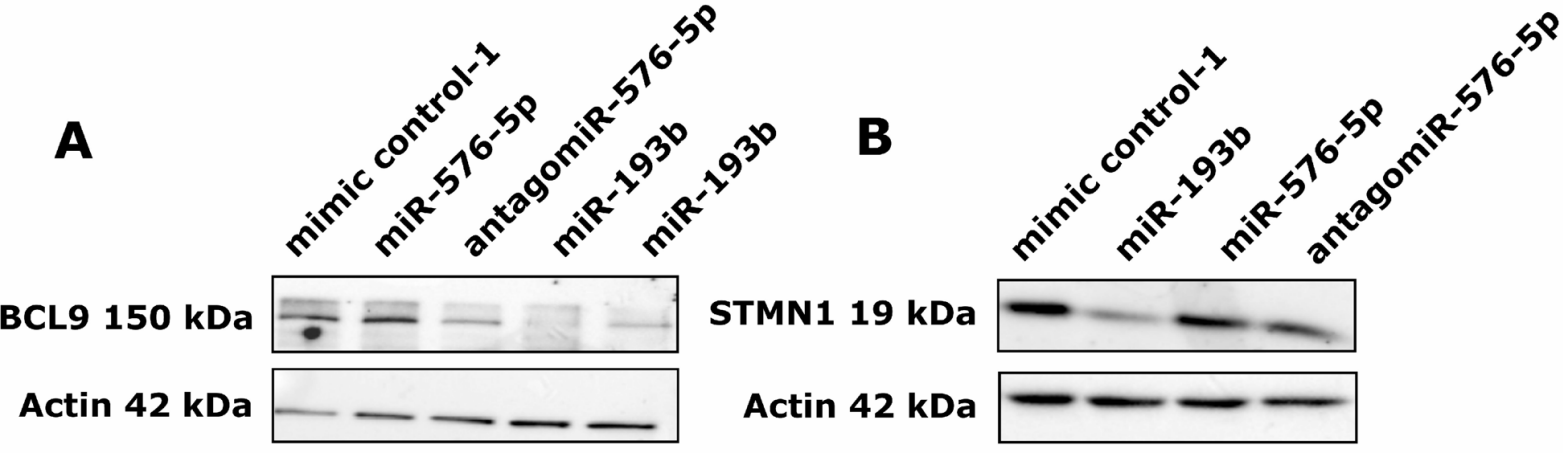
miR-193b downregulates protein expression of BCL9 and STMN1 A375 cells were transfected with 50 nM miRNA and 72 h post transfection cells were harvested and cellular protein was isolated. (**A**) For both miR-193b transfected samples, a clear reduction of BCL9 protein levels could be observed (FC = 0.09, FC = 0.20). Cells transfected with antagomiR-576-5p showed a strong decrease in BCL9 protein levels as well (FC = 0.30). (**B**) STMN1 protein levels were strongly reduced after miR-193b transfection (FC = 0.35) but remained unaltered after transfection with miR-576-5p or its antagomiR.

Downregulation of BCL9 expression by miR-193b could also be confirmed in A375 cells on protein level (FC = 0.09, FC = 0.20; Figure 7A). Moreover, cells transfected with antagomiR-576-5p showed a strong decrease in BCL9 protein levels as well (FC = 0.30). This observation is in accordance with the increased BCL9 mRNA expression levels observed in samples from miR-576-5p transfected A375 cells (Figure 6A). In line with our qPCR data (Figure 6D), protein expression of STMN1 was selectively downregulated upon transfection with miR-193b (FC = 0.35; Figure 7B).

**Figure 8:**
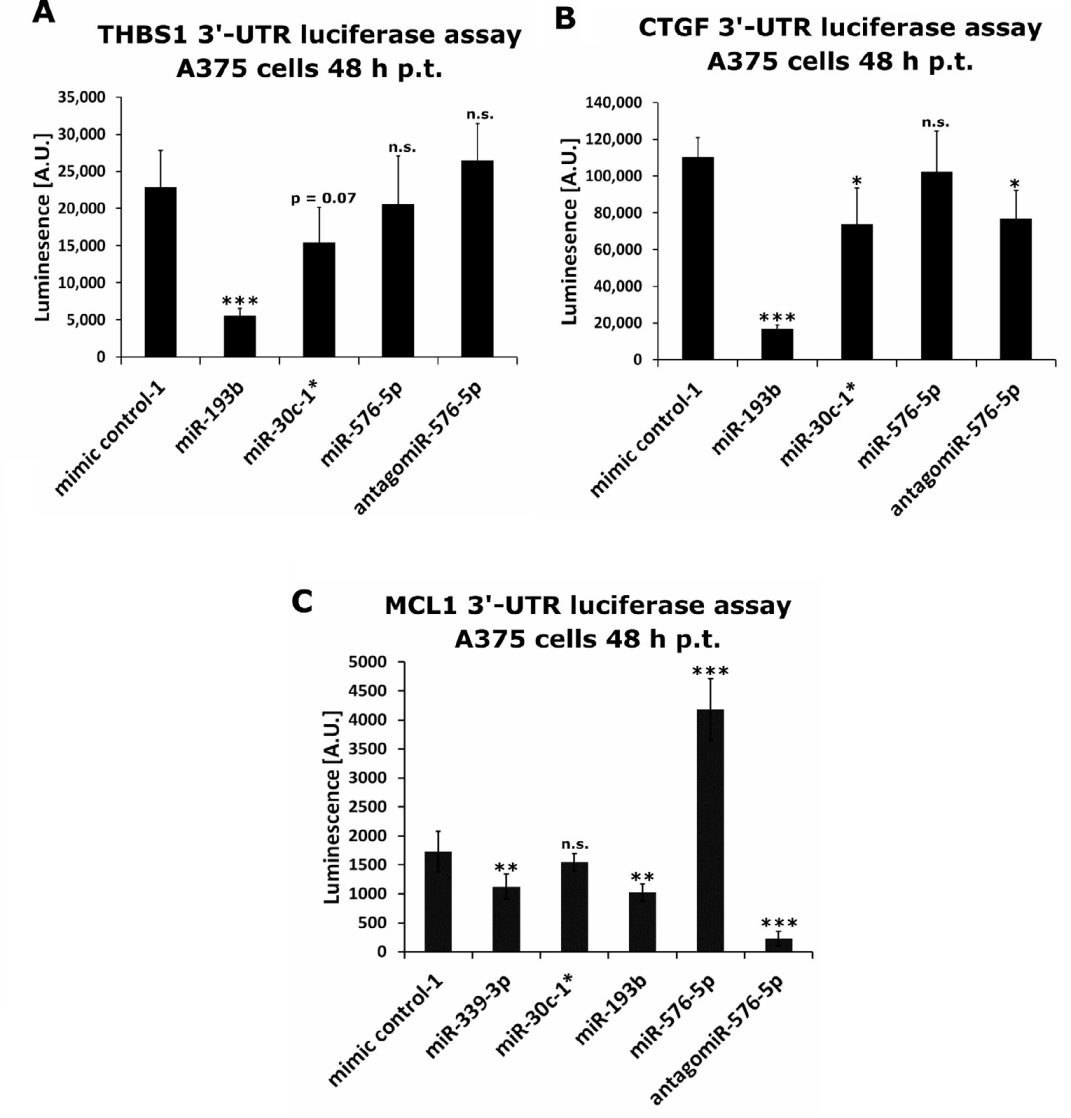
Luciferase based reporter assays performed on identified miRNA targets A375 cells were co-transfected with miRNA mimics or antagomiR together with a renilla luciferase reporter plasmid containing the 3′-UTR of THBS1 (**A**), CTGF (**B**) or MCL1 (**C**). Luminescence was measured 48 h post transfection. At least four biological replicates were performed per condition and mean values ± SD are shown. Conditions were compared to mimic control-1 transfections. Significance was assessed with two-sample Student’s *T*-tests. ^*^p < 0.05, ^**^p < 0.01, ^***^p < 0.005.

Next, the effect of miR-30c-1^*^, miR-193b and miR-576-5p on expression of CTGF, MCL1 and THBS1 was verified by luciferase reporter assays performed with A375 cells. Therefore, A375 cells were co-transfected with miRNA and an expression vector encoding the renilla luciferase gene fused to the 3′-UTR of the target gene of interest. The THBS1-specific luminescence signal was reduced after transfection with miR-30c-1^*^ (FC = 0.67) or with miR-193b (FC = 0.24), respectively (Figure 8A). Transfection with miR-576-5p or its antagomiR did not change the luminescence signal significantly. Of note, the CTGF-specific luminescence signal was also reduced after transfection with miR-30c-1^*^ (FC = 0.67) or miR-193b (FC = 0.15), respectively. Transfection with miR-576-5p did not result in a significant change of the luminescence signal, whereas transfection with its antagomiR significantly reduced the CTGF-specific luminescence signal (FC = 0.70; Figure 8B). The MCL1-specific luminescence signal was significantly reduced by miR-193b (FC = 0.59) and miR-339-3p (FC = 0.65), respectively, and also by antagomiR of miR-576-5p (FC = 0.13). Interestingly, the MCL1-specific luminescence signal was strongly increased after miR-576-5p transfection (FC = 2.42). We verified this result in an additional independent assay (see Supplementary Figure 7) observing the same effect of miR-576-5p and its antagomiR. This pronounced effect of miR-576-5p on the MCL-specific signal intensity became evident within 24 h after transfection, but reached its maximum after 48 h; of note, transfection of the antagomiR caused the opposite effect (see Supplementary Figure 8). These results were confirmed in HeLa cells showing the same enhancing effect of miR-576-5p on the MCL-1-specific luminescence signal intensity (see Supplementary Figure 9).

In summary we conclude that miR-30c-1^*^ and miR-193b function via direct down-regulation of their targets, whereas miR-576-5p acts contrarily, most likely by increasing target expression indirectly, e.g. by blockage of other miRNAs normally targeting the respective gene.

## DISCUSSION

The aim of this study was to further investigate a set of three miRNAs miR-30c-1^*^, miR-193b and miR-576-5p determined previously to affect invasiveness of human melanoma cell line A375 [7]. We could verify the accelerating effect of miR-576-5p as well as the inhibiting effects of miR-193b and miR-30c-1^*^ on invasion of A375 cells and were able to extend these findings to further human melanoma cell lines. Among the panel of melanoma cell lines tested, MaMel-103b turned out unsuitable for detection of miRNAs with inhibiting effects on invasion, most likely due to minimal invasive capacity *per se*.

Possible effects of the transfected miRNAs on the viability of the melanoma cells during the invasion assays could be ruled out by viability assays showing constant vitality of melanoma cells even 72 h after miRNA transfection. We thus conclude that the effects we observed in our invasion assay were due to progression to a more invasive phenotype after miR-576-5p transfection or to a less invasive phenotype after miR-30c-1^*^ and miR-193b transfection, respectively, rather than caused by impaired viability or reduced proliferative capacity.

**Figure 9:**
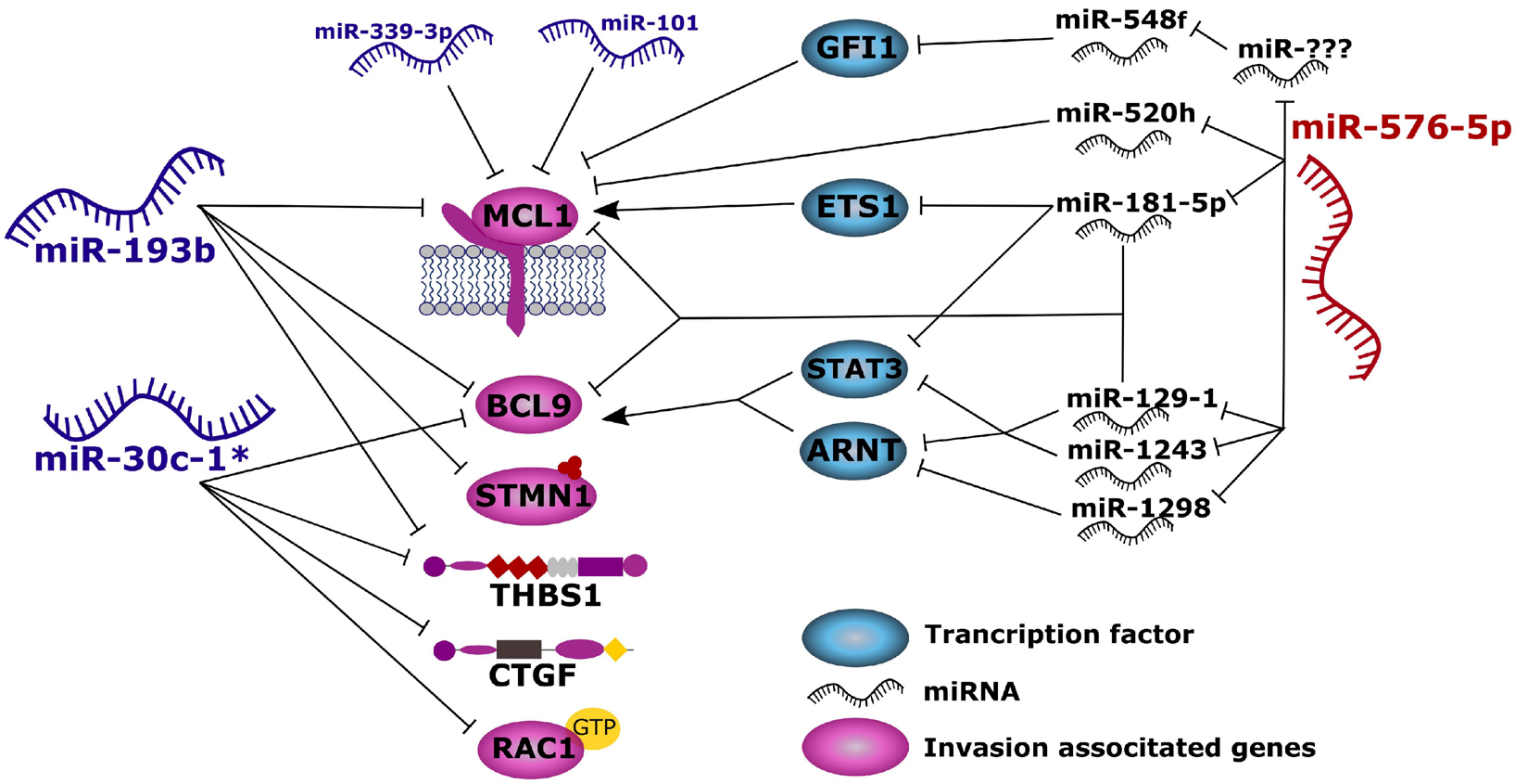
Proposed model summarizing the results of this study Anti-invasive miRNAs miR-193b and miR-30c-1^*^ act directly to the ^3′^-UTRs of invasion associated genes thereby blocking their translation. On the other hand, miR-576-5p promotes melanoma cell invasion through indirect up-regulation of MCL1 or BCL9, most likely by down-regulating other miRNAs.

Genomic profiling after transfection of miR-30c-1^*^, miR-193b and miR-576-5p revealed a set of genes allowing discrimination between highly and weakly invasive phenotypes. Based on the bioinformatic analyses and on our functional validation data, we propose a model introducing these three miRNAs as modulators within a functional network regulating translation of the targets identified in this study (Figure 9). We observed reduced CTGF and THBS1 mRNA levels after transfection with miR-30c-1^*^ and miR-193b. Of note, in a study by Braig and coworkers CTGF was found overexpressed in malignant melanoma and cell lines with low CTGF levels showed reduced invasiveness. The authors furthermore demonstrated that expression of recombinant CTGF enhanced invasion of treated cells [43], thus supporting the notion that CTGF repressing miR-30c-1^*^ and miR-193b represent possible tumor suppressor miRNAs.

Downregulation and enhanced silencing of THBS1 expression was also described as a consequence of DNA methylation in melanoma [44]. THBS1 as well as CTGF are part of the TGF-β signaling pathway, which showed up in our gene enrichment analysis for all three miRNAs (see Supplementary Table 4). This pathway is considered as a potential target for cancer therapy because TGF-β signaling is involved in various processes like cell growth, apoptosis, extracellular matrix formation, motility and invasion [45]. In fact, the TGF-β signaling pathway is often dysregulated in cancer and can sustain tumor suppressive as well as tumor promoting processes [46].

Our expression analysis showed downregulated expression of BCL9 in melanoma cells transfected with miR-30c-1^*^ or miR-193b, while BCL9 expression was enhanced in miR-576-5p transfected cells. BCL9 functions as coactivator of β-catenin, and in fact, the Wnt/β-catenin pathway appears dysregulated in various cancer types [47]. Especially in melanoma, the Wnt signaling pathway is associated with higher metastatic potential [48] and lack of anti-tumor immunity [49]. Furthermore, Wnt signaling contributes to the stabilization of MITF, thereby sustaining this pathway in melanocytes and melanoma cells [50]. BCL9 has been described as a promoter of tumor progression and invasion in different tumor entities, such as colon carcinoma and multiple myeloma [41]. It appears conceivable that miR-576-5p might enhance invasion by an indirect up-regulation of BCL9, whereas miR-30c-1^*^ and miR-193b suppress BCL9 levels, thereby causing a poorly invasive phenotype.

Interestingly, in miR-576-5p transfected A375 cells, the vast majority of endogenous miRNAs was found downregulated (Supplementary Figure 10). We speculate that miR-576-5p exerts its effect on invasion indirectly via targeting of endogenous miRNAs. In fact, we determined genes encoding miR-129-1, miR-1243 and miR-1298 to be expressed at significantly lower level after miR-576-5p transfection. These miRNAs, in turn, can target STAT3 and ARNT (transcription factor binding site prediction program from Qiagen) which represent transcription factors of BCL9, thus repression of these miRNAs through miR-576-5p might also lead to enhanced BCL9 expression levels. Indirect effects mediated by miR-576-5p could also explain the observed upregulation of the MCL1-associated luminescence signal in the ^3′^-UTR reporter assay. MCL1 is involved in melanoma cell survival and its expression is increased in melanoma cells compared to primary melanocytes. In melanoma, BRAF mutations can result in enhanced MCL1 levels, thereby increasing resistance to apoptosis [51]. Furthermore, MCL1 promotes epithelial mesenchymal transformation [52] and downregulation of MCL1 expression by siRNA reduced invasion of human melanoma cell lines, comparable to the anti-invasive effect described for miR-339-3p and miR-193b, both of which representing regulators of MCL1 expression [7, 53].

The direct interaction of miR-193b and miR-339-3p with MCL1 was confirmed in this study by ^3′^-UTR luciferase reporter-assays. Chen *et al.* already showed a direct regulation of MCL1 by miR-193b [53]. Other authors observed increased MCL1 expression induced by oncogenic BRAF in melanoma [51], thereby inducing a more aggressive phenotype. We speculate that miR-576-5p can enhance expression of MCL1 through an indirect mechanism, thus mimicking the effect of an oncogenic BRAF mutation. This might account for the highly-invasive phenotype observed in melanoma cells transfected with miR-576-5p. For example, miR-576-5p led to significantly decreased expression levels of genes encoding miR-181, miR-520h and miR-129-1. miR-520h and miR-129-1 have predicted binding sites for the seed sequences within the in ^3′^-UTR of MCL1. By downregulation of these miRNAs, their inhibiting effect on MCL1 might become weakened leading to higher MCL1 levels. miR-181-5p has been shown to target ETS1 which functions as positive transcriptional regulator of MCL1 [54]. Therefore, downregulation of miR-181-5p by miR-576-5p will increase ETS1 levels resulting in enhanced MCL1 expression. Among the few endogenous miRNAs upregulated in miR-576-5p transfected melanoma cells, we detected miR-548f which targets GFI1, representing a transcriptional repressor of MCL1 [55]. Possibly one or several of the downregulated miRNAs would target miR-548f under physiological conditions which could explain elevated levels of miR-548f in miR-576-p transfected melanoma cells. It therefore seems conceivable that miR-576-5p might alter the invasive capacity of human melanoma cells through a complex network of indirect interactions.

## MATERIALS AND METHODS

Additional methods are described in the Supplement. Antibodies and primer sequences are given in Supplementary Tables 1 and 2, respectively. The workflow for the gene expression profiling is depicted in Supplementary Figure 1.

### Cell lines and cell culture

Melanoma cell lines MaMel-86b and MaMel-103b used in this study were derived from metastases of patients with stage III or IV melanoma [25, 26]. A375 cells were purchased from ATCC. All melanoma cell lines were cultured in RPMI 1640 medium (Gibco, Carlsbad, CA) supplemented with 10 % FBS superior (Biochrom, Berlin, Germany) without antibiotics at 37° C and 5 % CO_2_. Authentication of cell lines was done by STR sequence analysis performed by the Forensic Medicine Department of the Heidelberg University Hospital (Heidelberg, Germany). Cell lines used to determine basal invasion levels are described in the supplement.

### Invasion assay

To measure invasiveness of human melanoma cell lines, 1·10^5^ cells per well were cultured in 24-well plates for 24 h followed by transfection with 50 nM miRNA. After 24 h, the culture medium was replenished. On the same day, HTS Transwell-96 well plates with a pore size of 8.0 μm (Corning), were coated with 10 µg/well Matrigel (Corning, Corning, NY) and dried overnight. After 48 h, cells harvested, were resuspended in medium without serum (FBS) and 50 μl cell suspension containing 5·10^4^ melanoma cells were added on the coated inlay membranes of the transwell plates. Three technical replicates were performed per condition. To the outer well, 150 μl medium with FBS were added serving as chemo-attractant. After incubation for 24 after at 37° C invasion was quantified. Therefore, calcein AM was dissolved in Dimethylsulfoxide (DMSO) and added to 1× cell dissociation solution (CDS, Trevigen, Gaithersburg, MD). Medium was removed from the outer compartment of the transwell plate. After washing with PBS, 100 μl 1× CDS-Calcein was pipetted in each well of black receiver plate (Corning). The inlay from the transwell plate was transferred carefully to the receiver plate. The plates were incubated for 1 h at 37° C and gentle agitation was performed every 15 minutes to ensure dissociation of cells attached to the lower side of the gel membrane. Fluorescence was measured using an Ascent Fluoroskan fluorometer (Thermo Fisher Scientific, Gothenburg, Sweden). Excitation wavelength was set to 485 nm, emission wavelength to 538 nm and integration time to 60 ms. To assess the correlation of miRNA expression levels and invasive capacity, 18 human melanoma cell lines were tested for their basal invasion level using the above described Boyden-chamber assay. One day after seeding 5·10^4^ cells per inner well in Boyden-chamber plates, invaded cells were fluorescently labeled and mean fluorescence intensity (MFI) was measured which is referred to as invasion score. For each cell line triplicates were performed.

## CONCLUSIONS

In conclusion, this study provides extended *in vitro* analyses of miR-576-5p and miR-193b and miR-30c-1^*^ on melanoma cell invasion as well as a selection of cellular genes predicted *in silico* as direct targets of these miRNAs. The miRNA mediated changes in gene expression were identified at transcriptional level by gene expression profiling and subsequent computational analysis. Several pathways and genes associated with invasion were identified. The expression levels of the identified target genes encoding CTGF, THBS1, STMN1, BCL9, RAC1 and MCL1 allowed discrimination between phenotypic groups of melanoma cell lines with high or low-invasive capacity, respectively. In melanoma, downregulation of miR-193b and miR-339-3p might occur as an early event in tumor progression. It appears conceivable that up-regulation of miR-576-5p might rescue the cancer cell from apoptosis, e.g. by up-regulation of MCL1 and BCL9, thereby increasing its invasive phenotype.

Our *in vitro* results suggest miR-576-5p to represent an oncomiR in melanoma, whereas miR-30c^*^ and miR-193b appear as potential tumor suppressors in this tumor entity. Utilization of suitable animal models including tumor cell lines with constitutive or inducible overexpression of these miRNAs will show whether their opposed function on tumor cell invasion is also reflected *in vivo*.

### Proliferation assay

Proliferation assays were performed using the impedance based xCELLigence system (ACEA Biosciences, San Diego, CA,) [27, 28]. In experiments assessing miRNA mediated effects on proliferation, A375 cells were reversely transfected upon addition of 1·10^4^ A375 cells per well to a 96 well E-plate that had been spotted with 50 nM miRNA per well beforehand (reverse transfection). Measurements were performed in 15 min intervals for 120 h. In order to assess proliferation effects which might infer with the invasion assay, a second xCELLigence assay was performed using 5·10^4^ cells per well. The assay was started 48 h after transfection and measurements were performed for 24 h.

### Luciferase-reporter assays

Cells were seeded in 96-well plates to achieve a confluency of 60–70 % at day of transfection. After 24 h, 50 nM miRNA and 100 ng/μl plasmid (pLS-MCL1-3′-UTR, pLS-CTGF- 3′-UTR or pLS-THBS1-3′UTR) were transfected using DharmaFect Duo (GE Dharmacon, Lafayette, CO) reagent. Two days post transfection cells were lysed with 100 μl Luciferase Cell Lysis Buffer per well. Plates were agitated for 20 min at 300 rpm at RT until lysis was complete. A working solution was prepared adding 50 μl 100× coelenterazine to 5 ml Renilla Glow Assay Buffer (Thermo Scientific, Boston, MA). Then, 50 μl working solution was added per well and luciferase activity was quantified with a Mithras luminescence reader. Plasmids containing 3′-UTRs of targets fused to the renilla luciferase gene were purchased from Switch Gear Genomics.

## SUPPLEMENTARY MATERIALS FIGURES AND TABLES




