# Supplementary material for: miR-193b and miR-30c-1* inhibit, whereas miR-576-5p enhances melanoma cell invasion in vitro
Source: Oncotarget. 2018 Aug 21;9(65):32507–22. doi: 10.18632/oncotarget.25986 (PMC6126698; doi:10.18632/oncotarget.25986)
Supplement: Supplementary file 1 [file oncotarget-09-32507-s001.pdf]

miR-193b and miR-30c-1* inhibit, whereas miR-576-5p enhances melanoma cell invasion *in vitro*

SUPPLEMENTARY MATERIALS

Melanoma cell lines

MaMel and UKRV cell lines were established from melanoma metastases and are described elsewhere [1–3]. The human primary melanoma cell line WM98.1 was kindly provided by Eva Frei (DKFZ). All cell lines used are listed in Supplementary Table 3.

Cell viability assay

Cell viability was assessed with the CellTiter-Glo Luminescent Cell Viability Assay (Promega, Fitchburg, WI, USA). Therefore, cells were cultured in black/clear flat bottom plates (Corning, Big Flats, NY, USA) seeding $1 \cdot 10^4$ to $2 \cdot 10^4$ cells per well, depending on proliferative activity. After 24 h, cells were transfected with 50 nM miRNA preparing three biological replicates per condition. Culture medium was replenished every 24 h. On third day post transfection cell viability was measured using a Fluoroskan ascent FL machine according to the manufacturer's protocol, using 60 ms as integration time.

XTT cell proliferation assay

To determine effect of miRNA transfection on proliferative effects in MaMel-86b and MaMel-103b which might interfere with the invasion assay, XTT proliferation assays (PromoKine, Heidelberg, Germany) were performed according the manufactures protocol. Cells were treated exactly as for the invasion assay.

Gene expression profiling and analysis

The basic work flow of the gene expression profiling is illustrated in Supplementary Figure 1. A375 cells were transfected with 50 nM miR-576-5p, miR-30c-1*, miR-193b or mimic control-1 (three biological replicates per condition). After 48 h, cells were harvested and RNA was isolated for gene expression profiling using an Illumina Human-HT 12 chip. Expression profiling, background correction, normalization and differential gene expression analysis was conducted by the Core Facility of the DKFZ. The data were grouped according to the biological replicates and comparison of expression levels was performed between melanoma cells transfected with miRNA of interest and cells transfected with mimic control-1. The expression data were $\log_2$ transformed and z-score normalization was performed gene-wise [4, 5].

Gene set enrichment analysis (GSEA) was performed using the R-package piano. For each miRNA, the corresponding genes with their respective *p*-values were loaded into the GSEA function. The statistical method was set to Fisher and the assessment of significance was performed by gene sampling with 1000 permutations. Furthermore, DAVID 6.7 was used to find enriched gene clusters. The differentially expressed genes ($p < 0.005$) were ranked by their expression fold changes. Fold changes (FC) greater 1.3 were considered as up-regulated, whereas FC less than 0.7 were considered as down-regulated. The gene lists for up- or down regulated genes, respectively were loaded into the DAVID online platform for each miRNA. The background gene list was set to Illumina Human HT-12.

qPCR

mRNA expression was analyzed by Taqman qPCR. RNA was isolated from frozen cell pellets using the miRNeasy Mini Kit (Qiagen, Hilden, Germany) according to the manufacturer's protocol. 500 ng total RNA were reversely transcribed using the Transcriptor First Strand cDNA Synthesis Kit (Roche Applied Science, Mannheim, Germany) in a total volume of 20 µl. For qPCR analysis, 2 µl of diluted (1:5) cDNA solution were applied in a total volume of 20 µl using TaqMan universal PCR Mastermix (Applied Biosystems, Foster City, CA, USA). GAPDH was used as housekeeping gene. For all miRNAs tested for impact on mRNA expression level, prediction of miRNA binding to the 3′-UTR of GAPDH was performed to test whether GAPDH is a suitable housekeeping gene. None of the investigated miRNAs exhibited a predicted binding site. For cDNA synthesized from miRNA using the Taqman miRNA assay kit (Applied Biosystems), 2 µl (16 ng) were used in a 20 µl PCR reaction volume and small nuclear RNA U6 (RNU6B) was used as endogenous control. All taqman probes were purchased from Applied Biosystems. For all samples, three technical replicates

were performed and relative expression was calculated with the 2−ΔΔCt-method according to the manufacturer's protocol.

Western blot analysis

After transfection, protein concentrations within lysates of frozen cells were measured with Pierce BCA protein assay kit according to the manufacture's protocol. Lysates were heat denatured for 5 min at 96° C and cellular extracts containing 15 µg protein per lane were loaded onto polyacrylamide gels (10 or 12%). After electro-transfer of separated protein extracts, nitrocellulose membranes were blocked with 5% BSA for 1 h at RT. Primary antibodies were incubated at 4° C o/n in blocking solution. After extensive washing, membranes were incubated for 1 h at RT with the corresponding secondary antibodies coupled to horse reddish peroxidase. Protein signals were then detected with ECL-Prime reagent (GE Healthcare) and measurements were performed on a ChemiDoc XRS system (Bio-Rad). Densitometric quantification of protein bands was performed using ImageLab software. Actin was used as loading control and signal intensities of proteins bands were normalized to actin. All antibodies used are listed in Supplementary Table 1.

Statistical analysis

All statistical analyses were performed using R version 3.01.1 or Microsoft Excel 14.0. Pearson's Correlation Coefficient (PCC) was used to determine correlation of miRNA expression and invasiveness of human melanoma cell lines. To assess significance, two-sided, two-sample Student's *T*-Tests were performed and *p*-values smaller 0.05 were considered as statistically significant.

Network construction and miRNA binding site prediction

To generate Figure 9 we used Inkscape and the tool miRmap to evaluate miRNA binding sites in respective target gene 3′-UTRs [6].

Supplementary Spread Sheet 1: Differential_Genes_1

REFERENCES

1. Hoek KS, Eichhoff OM, Schlegel NC, Dobbeling U, Kobert N, Schaerer L, Hemmi S, Dummer R. *In vivo* switching of human melanoma cells between proliferative and invasive states. Cancer Res. 2008; 68:650–656.
2. Hoek KS, Schlegel NC, Brafford P, Sucker A, Ugurel S, Kumar R, Weber BL, Nathanson KL, Phillips DJ, Herlyn M, Schadendorf D, Dummer R. Metastatic potential of melanomas defined by specific gene expression profiles with no BRAF signature. Pigment Cell Res. 2006; 19:290–302.
3. Tang XR, Wen X, He QM, Li YQ, Ren XY, Yang XJ, Zhang J, Wang YQ, Ma J, Liu N. MicroRNA-101 inhibits invasion and angiogenesis through targeting ITGA3 and its systemic delivery inhibits lung metastasis in nasopharyngeal carcinoma. Cell Death Dis. 2017; 8:e2566.
4. Cheadle C, Vawter MP, Freed WJ, Becker KG. Analysis of Microarray Data Using Z Score Transformation. The Journal of Molecular Diagnostics. 2003; 5:73–81.
5. Lazar C, Meganck S, Taminau J, Steenhoff D, Coletta A, Molter C, Weiss-Solís DY, Duque R, Bersini H, Nowé A. Batch effect removal methods for microarray gene expression data integration: a survey. Briefings in Bioinformatics. 2013; 14:469–490.
6. Vejnar CE, Zdobnov EM. MiRmap: comprehensive prediction of microRNA target repression strength. Nucleic Acids Res. 2012; 40:11673–11683.
7. Sun Y, Moller P, Berking C, Schlupen EM, Volkenandt M, Schadendorf D. *In vivo* selective expansion of a tumour-specific cytotoxic T-cell clone derived from peripheral blood of a melanoma patient after vaccination with gene-modified autologous tumour cells. Immunology. 1999; 98:535–540.

Supplementary Table 1: Antibodies used for Western blot analysis

Antibody	Type	Manufacturer
Anit-Actin clone C4 human	Mouse monoclonal	MP Biomedicals, Illkirch, France
Goat anti-mouse sc2005	IgG-HRP	Santa Cruz Biotechnology, Heidelberg, Germany
Goat anti-rabbit sc2004	IgG-HRP	Santa Cruz Biotechnology, Heidelberg, Germany
BCL9 #15096	Rabbit polyclonal	Cell Signaling, Cambridge, UK
STMN1 Op18 (G-8) sc-48362	Mouse monoclonal	Santa Cruz Biotechnology, Heidelberg, Germany

Supplementary Table 2: Sequence of miRNAs used in this study

miRNA	Sequence 5′-3′	Purpose
**hsa-miR-101**	CAGUUAUCACAGUGCUGAUGCU	control invasion assay
**hsa-miR-137**	UUAUUGCUUAAGAAUACGCGUAG	control proliferation assay
**hsa-miR-182**	UUUGGCAAUGGUAGAACUCACACU	control invasion assay
**hsa-miR-193b**	CGGGGUUUUGAGGGCGAGAUGA	candidate miRNA
**hsa-miR-30c-1***	CUGGGAGAGGGUUGUUUACUCC	candidate miRNA
**hsa-miR-339-3p**	UGAGCGCCUCGACGACAGAGCCG	candidate miRNA
**hsa-miR-576-5p**	AUUCUAAUUUCUCCACGUCUUU	candidate miRNA

Supplementary Table 3: Invasive capacity of 18 human melanoma cell lines

Cell line	MFI [A.U.] = Invasion score	SD
**MaMel-79b**	0.494	0.0064
**MaMel-51**	0.589	0.0333
**MaMel-61e**	0.603	0.0302
**MaMel-68**	0.903	0.0354
**MaMel-20**	0.910	0.0245
**MaMel-36**	1.034	0.0835
**MaMel-21**	1.203	0.0178
**MaMel-86b**	1.318	0.1107
**UKRV-Mel-17**	1.326	0.0334
**MaMel-37b**	1.515	0.0429
**MaMel-73a**	1.640	0.0622
**UKRV-Mel-21**	2.609	0.0588
**MaMel-05**	2.873	0.0540
**MaMel-103b**	2.895	0.0254
**MaMel-57**	3.236	0.1838
**A375**	3.326	0.0369
**WM 98.1**	4.766	0.1035
**MaMel-19**	4.922	0.4514

One day after seeding $5 \cdot 10^4$ cells per inner well in Boyden-chamber plates, invaded cells were fluorescently labeled and mean fluorescence intensity (MFI) was measured which is referred to as invasion score. For each cell line triplicates were performed.
A375 was purchased from ATCC. WM 98.1 were kindly provided by Dr. Eva Frei (DKFZ). All other cell lines (our laboratory) were generated from melanoma samples as described elsewhere [1, 2, 7].

Supplementary Table 4: Enriched pathways after miRNA transfection of A375 cells

miR-193b	miR-30c-1*	miR-576-5p
Metabolic pathways	Metabolic pathways	Metabolic pathways
Cell cycle	Cell cycle	Pathogenic E.coli infection
Pathways in cancer	Pathways in cancer	Shigellosis
Viral myocarditis	Histidine metabolism	Adherens junction
Histidine metabolism	Phagosome	Rheumatoid arthritis
Rheumatoid arthritis	Focal adhesion	Phagosome
Phagosome	TGF-β signaling pathway	Focal adhesion
Focal adhesion	Oocyte meiosis	Bacterial invasion of epithelial cells
TGF-β signaling pathway	Tyrosine metabolism	TGF-β signaling pathway
Oocyte meiosis	Phenylalanine metabolism	Glycolysis
Pancreatic cancer	P53 signaling pathway	ECM-receptor interaction
Small cell lung cancer	RNA degradation	Spliceosome

Analysis was performed with GSEA which considers pathways that exhibit up- as well as down-regulated genes. Only pathways with adjusted p value < 0.01 are listed.

Supplementary Table 5: Enriched pathways after miRNA transfection of A375 cells

miR-193b		miR-30c-1*		miR-576-5p	
**Enriched pathways for down-regulated genes**					
**Pathway**	***p* value**	**Pathway**	***p* value**	**Pathway**	***p* value**
Negative regulation of cell proliferation	2.5E-4	Cell adhesion	4E-5	Cell adhesion	0.001
Regulation of organelle organization	4.7E-4	Biological adhesion	4E-5	Biological adhesion	0.001
Cell cycle	7.1E-4	Blood vessel morphogenesis	5E-5	Integrin binding	0.002
Positive regulation of cytoskeleton organization	0.001	Blood vessel development	1E-4	Transforming growth factor beta binding	0.01
Intracellular signaling cascade	0.001	Vasculature development	1.1E-4	Angiogenesis	0.01
Growth factor binding	0.002	Positive regulation of cell adhesion	2.1E-4	Cell motion	0.02
Response to calcium ion	0.002	Cell motion	2.7E-4	Response to wounding	0.02
Positive regulation of cell adhesion	0.003	Heparin binding	8.1E-4	Pattering of blood vessels	0.03
**Enriched pathways for up-regulated genes**					
**Pathway**	***p* value**	**Pathway**	***p* value**	**Pathway**	***p* value**
Hemostasis	5.3E-4	Regulation of smooth muscle cell proliferation	0.003	Extracellular region	0.002
Regulation of body fluid levels	7E-4	Negative regulation of smooth muscle cell proliferation	0.004	Cell proliferation	0.006
Response to wounding	8.3E-4	Regulation of cell proliferation	0.005	Membrane-bounded vesicle	0.03
Blood coagulation	0.001	Extracellular matrix part	0.007	Growth factor binding	0.03
Coagulation	0.002	Perinuclear region of cytoplasm	0.01	Cytokine binding	0.03
Wound healing	0.02	Symporter activity	0.01	Cytokine receptor activity	0.05

Analysis was performed using online tool DAVID. As a result of the DAVID pathway analysis, differentially expressed genes with a FC smaller than 0.7 or higher than 1.3 respectively, are shown.

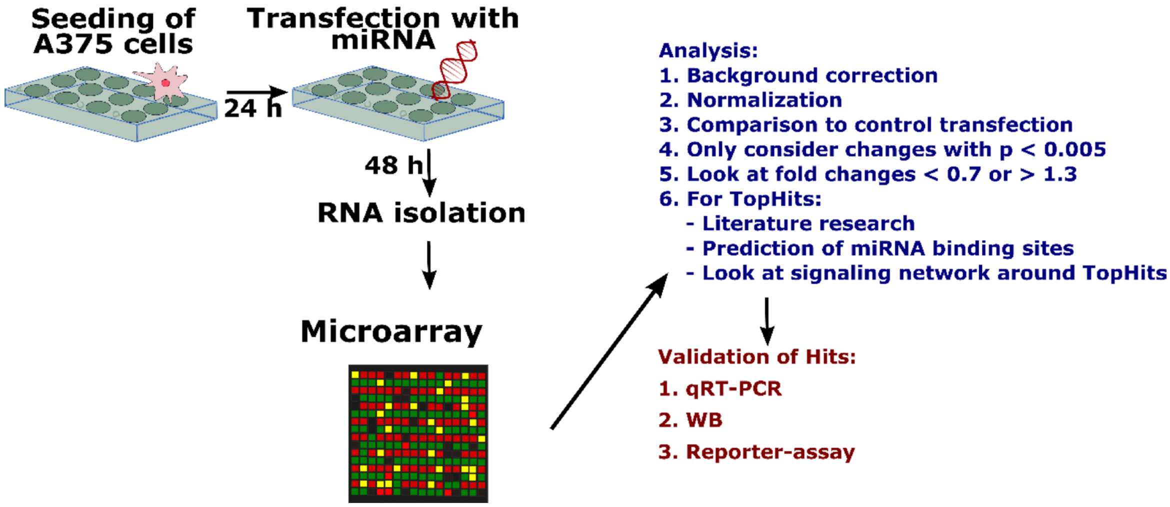

Supplementary Figure 1: Workflow of gene expression profiling after miRNA transfection. A375 cells were transfected with 50 nM mimic control-1, miR-576-5p, miR-193b or miR-30c-1*. Two days post transfection, total RNA was isolated and used for gene expression profiling on an Illumina Human-HT 12 chip. Three biological replicates were performed per condition. Effects of miRNA samples were compared to mimic control-1 samples.

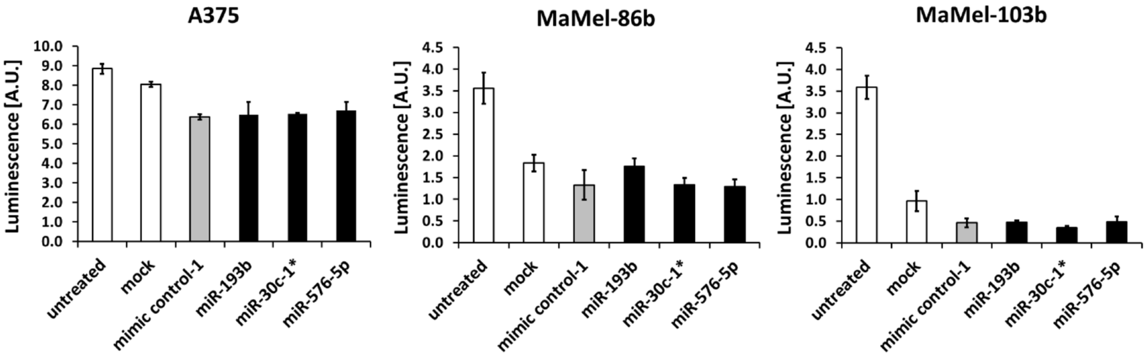

Supplementary Figure 2: miR-193b, miR-30c-1* and miR-576-5p do not affect viability of various human melanoma cell lines. Viability of melanoma cells lines transfected with 50 nM miRNA was measured 72 h post transfection. None of the miRNAs showed a significant effect on cell viability compared to mimic control-1 transfected samples. Three biological replicates were performed per condition and mean values ± SD are displayed. Significance was assessed by two-sample Student's *T*-test.

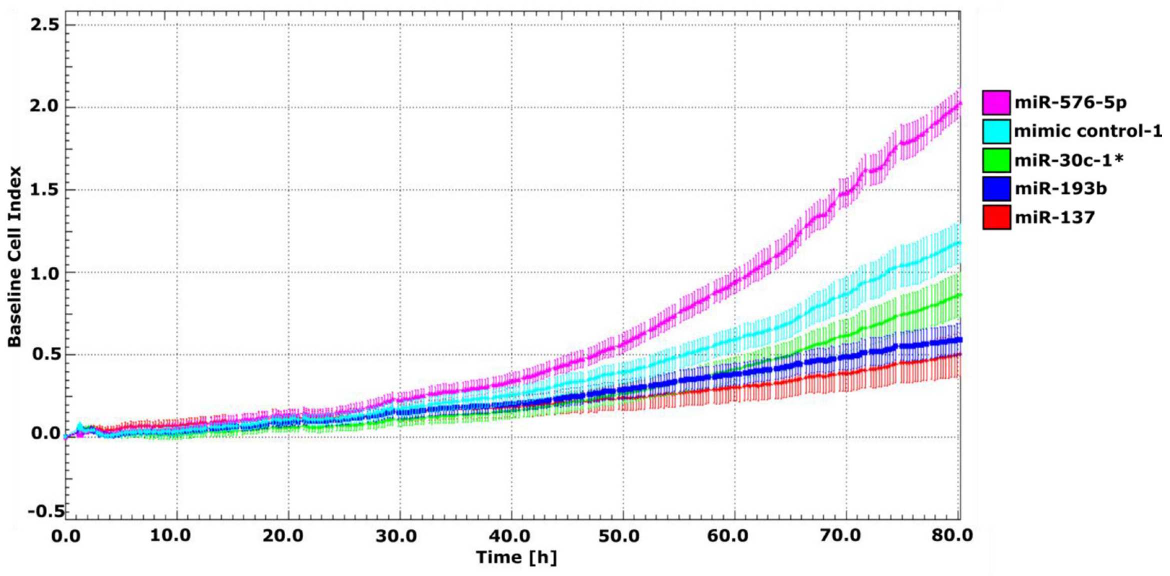

Supplementary Figure 3: Proliferation assay using XCELLigence system. A375 cells were reversely transfected with 50 nM miRNA in E-plates and proliferation was monitored by impedance measurement which is correlated with cell number and expressed as Baseline Cell Index. Samples transfected with miR-137 served as a control for reduced proliferation. Three biological replicates were performed per condition and mean values ± SD are displayed. Transfection with miR-576-5p enhanced proliferation compared to transfection with mimic control-1, whereas miR-137, miR-193b as well as miR-30c-1* reduced the proliferation rate of A375 cells. These effects become evident approx. 48 h after transfection.

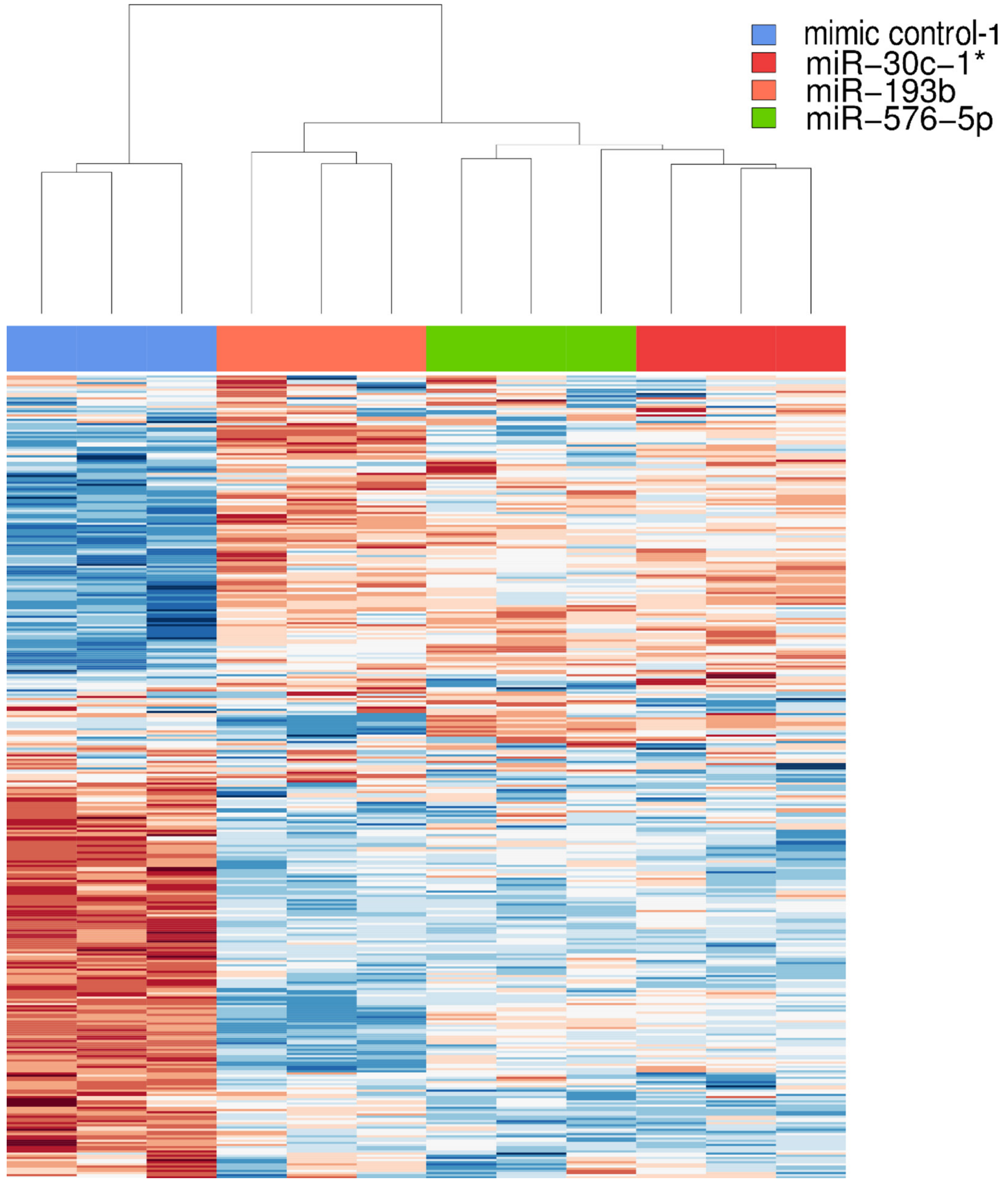

Supplementary Figure 4: Heatmap and cluster analysis for A375 cells after miRNA transfection. Gene expression data was $\log_2$ transformed and z-normalized. For the clustering based on average linkage, 371 genes were used which were differentially expressed for all three miRNAs. Red represents low expression, white average expression and blue high expression. The three biological replicates for each condition cluster together. However, the two groups of high and low-invasive potential do not cluster together.

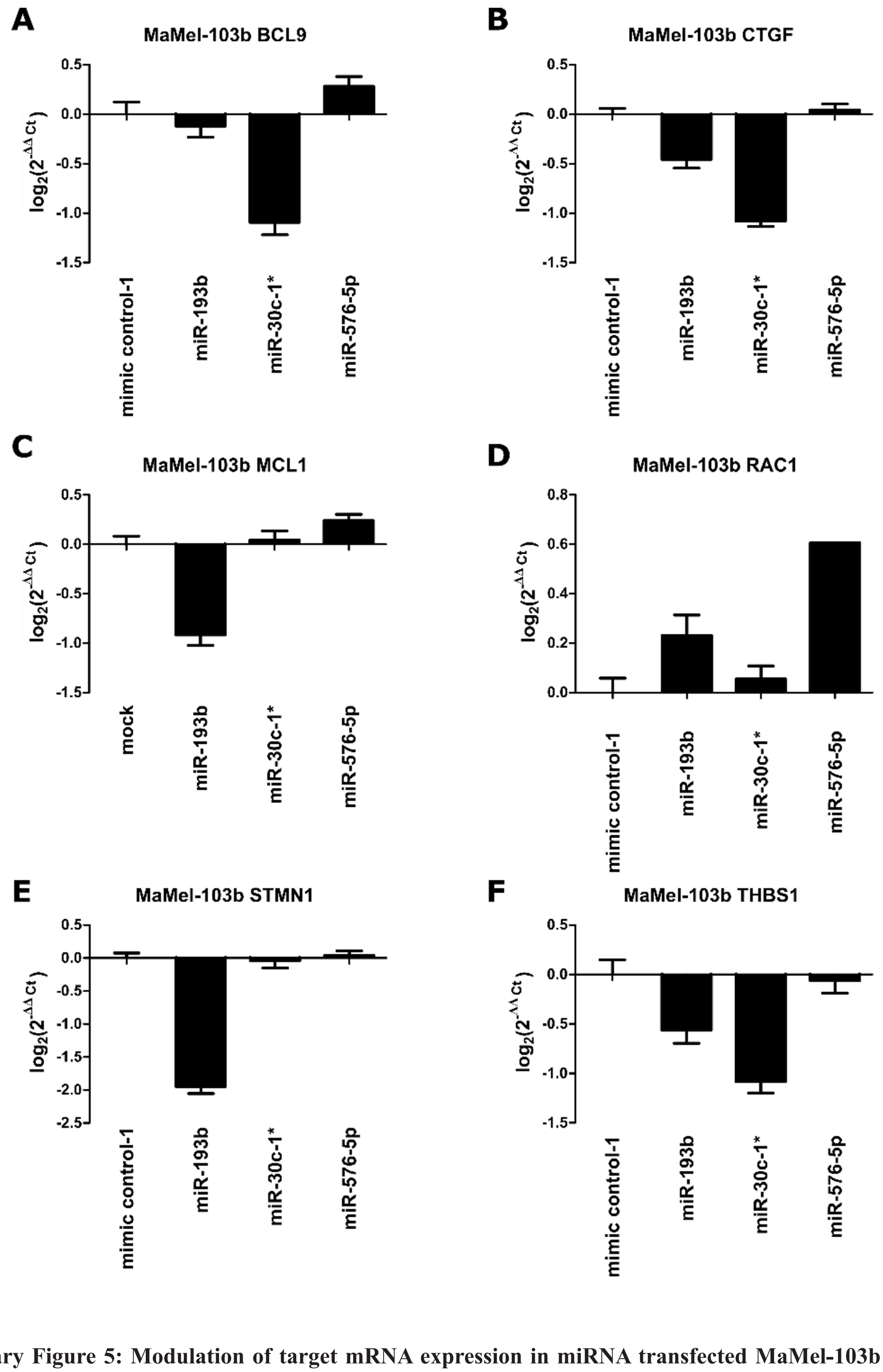

Supplementary Figure 5: Modulation of target mRNA expression in miRNA transfected MaMel-103b cells. MaMel-103b cells were transfected with 50 nM mimic control-1, miR-30c-1*, miR-193b or miR-576-5p. After RNA isolation, cDNA was generated and qPCR was performed. Expression levels of BCL9 **(A)**, CTGF **(B)**, MCL1 **(C)**, RAC1 **(D)**, STMN1 **(E)** and THBS1 **(F)** were normalized to GAPDH. Mean values of three technical replicates are shown ± SD. Conditions were compared to mimic control-1. Only for MCL1 miRNA transfected samples were compared to mock transfections since mimic control-1 exhibits two bindings sites within the MCL1 3′-UTR based on miRmap.

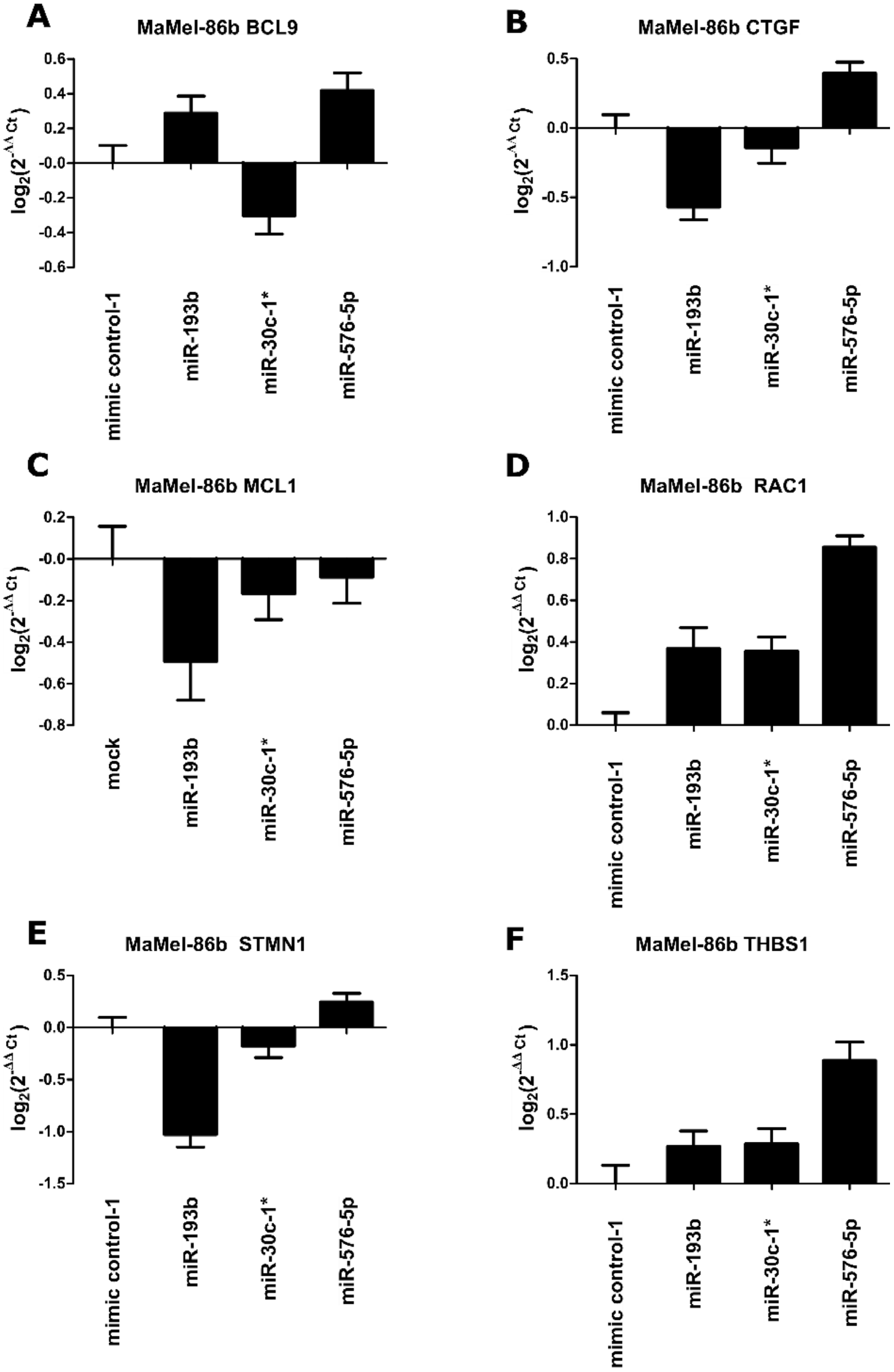

Supplementary Figure 6: Modulation of target mRNA expression in miRNA transfected MaMel-86b cells. MaMel-86b cells were transfected with 50 nM mimic control-1, miR-30c-1*, miR-193b or miR-576-5p. After RNA isolation, cDNA was generated and qPCR was performed. Expression levels of BCL9 **(A)**, CTGF **(B)**, MCL1 **(C)**, RAC1 **(D)**, STMN1 **(E)** and THBS1 **(F)** were normalized to GAPDH. Mean values of three technical replicates are shown ± SD. Conditions were compared to mimic control-1. Only for MCL1 miRNA transfected samples were compared to mock transfections since mimic control-1 exhibits two bindings sites within the MCL1 3′-UTR based on miRmap.

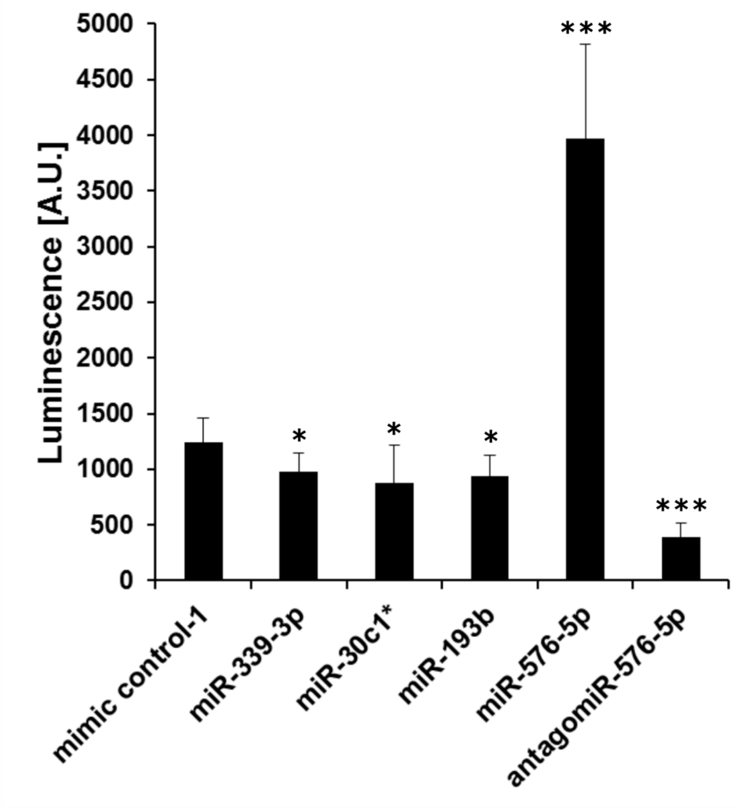

Supplementary Figure 7: MCL-1 3′-UTR reporter assay. A375 cells were transfected with 50 nM miRNA and 100 ng pLS-MCL1 containing the 3′-UTR of MCL1 fused to the renilla luciferase gene. Luminescence was measured 48 h post transfection. Seven biological replicates were performed per condition and mean values ± SD are shown. Conditions were compared to mimic control-1 and significance was assessed with two-sample Student's *T*-tests. $^{*}p < 0.05$, $^{**}p < 0.01$, $^{***}p < 0.005$.

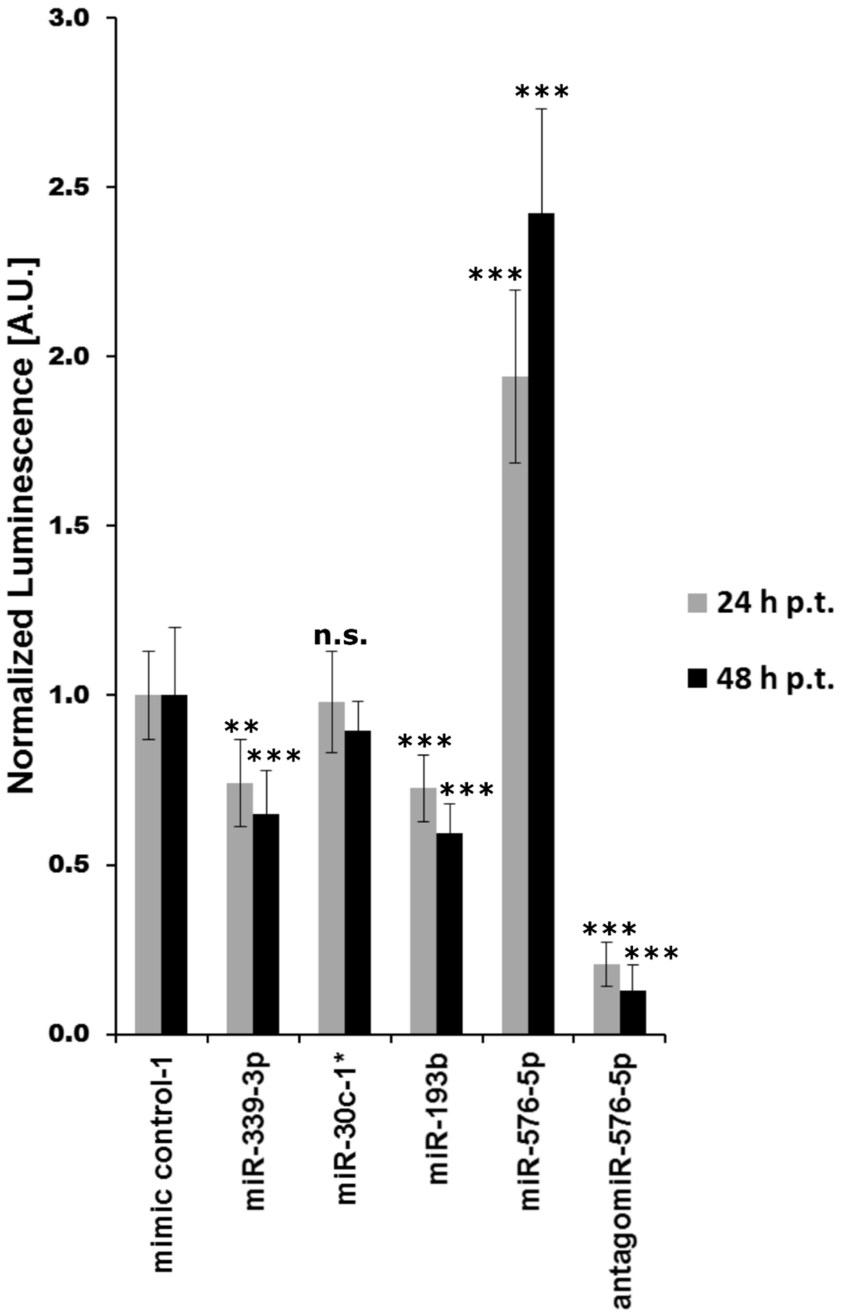

Supplementary Figure 8: MCL-1 3′-UTR reporter assay in A375 cells. A375 cells were transfected with 50 nM miRNA and 100 ng pLS-MCL1 containing the 3′-UTR of MCL1 fused to the renilla luciferase gene. Luminescence was measured 24 h and 48 h post transfection. Seven biological replicates were performed per condition and mean values ± SD are shown. Conditions were compared to mimic control-1 and significance was assessed with two-sample Student's *T*-tests. $^{*}p < 0.05$, $^{**}p < 0.01$, $^{***}p < 0.005$.

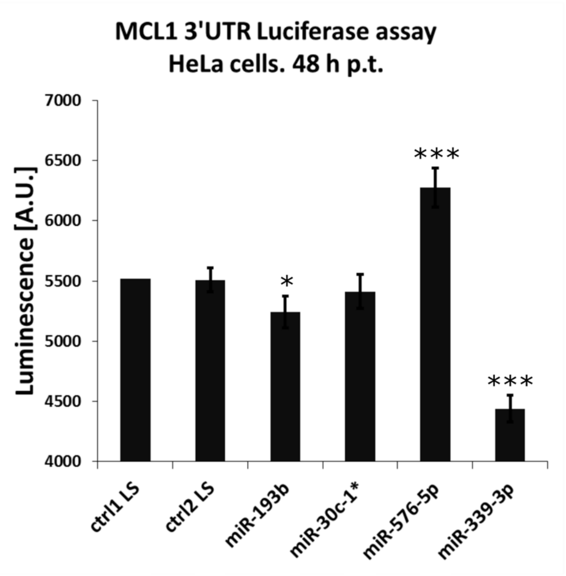

Supplementary Figure 9: MCL-1 3′-UTR reporter assay in HeLa cells. HeLa cells were transfected with 50 nM miRNA and 100 ng pLS-MCL1 containing the 3′-UTR of MCL1 fused to the renilla luciferase gene. Luminescence was measured 48 h post transfection. Four biological replicates were performed per condition and mean values ± SD are shown. Conditions were compared to mimic control ctrl2 LS. Significance was assessed with two-sample Student's *T*-tests. $^{*}p < 0.05$, $^{**}p < 0.01$, $^{***}p < 0.005$.

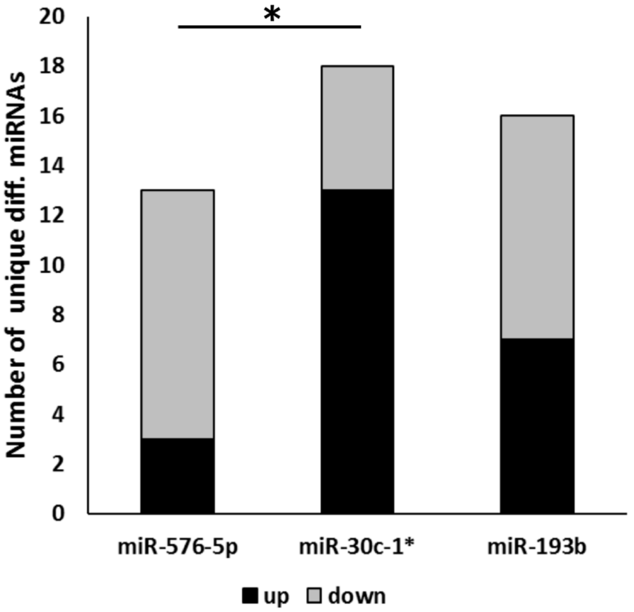

Supplementary Figure 10: Number of differentially expressed miRNA genes. A375 cells were transfected with 50 nM miRNA and gene expression profiling was performed. For each miRNA the number of differentially expressed miRNA genes was determined in comparison to mimic control-1 transfected samples. Only those genes uniquely altered by either miR-576-5p, miR-30c-1* or miR-193b were counted. Interestingly, miR-576-5p transfected samples showed significantly less up-regulated miRNAs compared to miR-30c-1*. Significance was assessed with Fisher Exact Test. $^{*}p < 0.05$.
